# Leg length discrepancy: A systematic review on the validity and reliability of clinical assessments and imaging diagnostics used in clinical practice

**DOI:** 10.1371/journal.pone.0261457

**Published:** 2021-12-20

**Authors:** Martin Alfuth, Patrick Fichter, Axel Knicker

**Affiliations:** 1 Faculty of Health Care, Therapeutic Sciences, Niederrhein University of Applied Sciences, Krefeld, Germany; 2 Department of Further Education, M.Sc. Sport Physiotherapy, German Sport University Cologne, Cologne, Germany; 3 Institute of Movement and Neurosciences, German Sport University Cologne, Cologne, Germany; Assiut University Faculty of Medicine, EGYPT

## Abstract

**Background:**

A variety of assessments to determine leg length discrepancy (LLD) is used in clinical practice and evidence about validity and reliability may differ.

**Objective:**

The objective of this systematic review was to identify and describe the validity and reliability of different assessments and imaging diagnostics for the determination of LLD.

**Materials and methods:**

The review was conducted following the recommendations of Preferred Reporting Items for Systematic reviews and Meta-Analyses (PRISMA). The databases Medline (PubMed) and Index to Chiropractic Literature were systematically searched. Studies regarding clinical assessments and imaging diagnostics for the diagnosis of LLD, which reported the clinimetric properties for assessment of LLD, were included and screened for methodological quality using the Quality Assessment of Studies of Diagnostic Accuracy (QUADAS-2) tool for validity studies and the Quality Appraisal of Diagnostic Reliability (QAREL) tool for reliability studies.

**Results:**

Thirty-seven articles on clinical assessments and 15 studies on imaging diagnostics met the eligibility criteria. Thirteen studies on the validity of clinical assessments and six studies on the validity of imaging diagnostics had a low risk of bias and low concerns regarding applicability for all domains. One study on the reliability of clinical assessments and one study on the reliability of imaging diagnostics had a low risk of bias. Main limitations were, that an analysis of sensitivity and specificity was only performed in a few studies and that a valid reference standard was lacking in numerous studies on clinical assessments.

**Conclusions:**

For the clinical assessment of LLD, the block test appears to be the most useful method. Full-length standing anteroposterior radiography seems to be the most valid and reliable method and may be used as global reference standard to measure the anatomic LLD when comparing clinical methods and imaging diagnostics.

## Introduction

Clinicians and physiotherapists are frequently confronted with leg length discrepancy (LLD) in their patients [1, 2]. LLD is categorized as an anatomic or functional type [3] and may result in multiple problems for the affected patient. It was declared that when joint pain in the lower extremities arises, any leg length discrepancy should be corrected to prevent increased stresses of weight bearing at other joints that may result in joint dysfunction from relatively harmless movements [4]. Some patients show both types of LLD, which can lead to a compensation or an increase of LLD. The anatomic LLD arises, when the cumulative bone length and the thickness of cartilage significantly differ between both legs. Gurney [5] states that the causes for the anatomic LLD are congenital or acquired. The most prevalent congenital ones are dislocation of the hip and hemiatrophy or hemihypertrophy with skeletal involvement of the lower extremity. Acquired causes may develop from infections, palsy, tumors, surgery, e.g. arthroplasty of the hip or knee, or slipped capital femoral epiphysis [5, 6]. Functional LLD may be caused by contracture of soft tissue, contractures of the hip or knee joints, pelvic obliquity or foot deformities [3, 7]. During loaded standing functional and anatomic LLD may interact.

The prevalence of anatomical LLD in the population is 90% [1]. Forty-one percent (41.3%) of the population demonstrate an anatomic LLD of 0–4 mm, 37.4% of 5–9 mm, 20% of more than 9 mm, 15% of 10–14 mm and 6.4% of more than 14 mm. LLD of > 5 mm is related to an increased risk of osteoarthritis of the hip and knee joints [8] as well as low back pain and lumbar scoliosis [9]. Moreover, a LLD > 6 mm is associated with an increased intensity of low back pain [10] and > 10 mm with an enhanced rate of hip and knee arthroplasties [9].

A valid, reliable and accurate method to measure leg length discrepancy is important to treat the patients effectively. A variety of assessments and imaging diagnostics to determine LLD is used, however, evidence about validity, reliability and diagnostic accuracy may differ [5, 11]. Moreover, it was concluded that the measurement of leg length discrepancy using a tape measure and standing on blocks is not as accurate as the measurement using imaging methods [12]. Therefore, the aim of this systematic review was to identify, describe, and compare the validity and reliability of different clinical assessments and imaging diagnostics for the measurement of LLD. It was hypothesized that imaging diagnostics are more valid, reliable, and accurate than clinical measurement methods.

## Materials and methods

### Study design and search strategy

**Fig 1:**
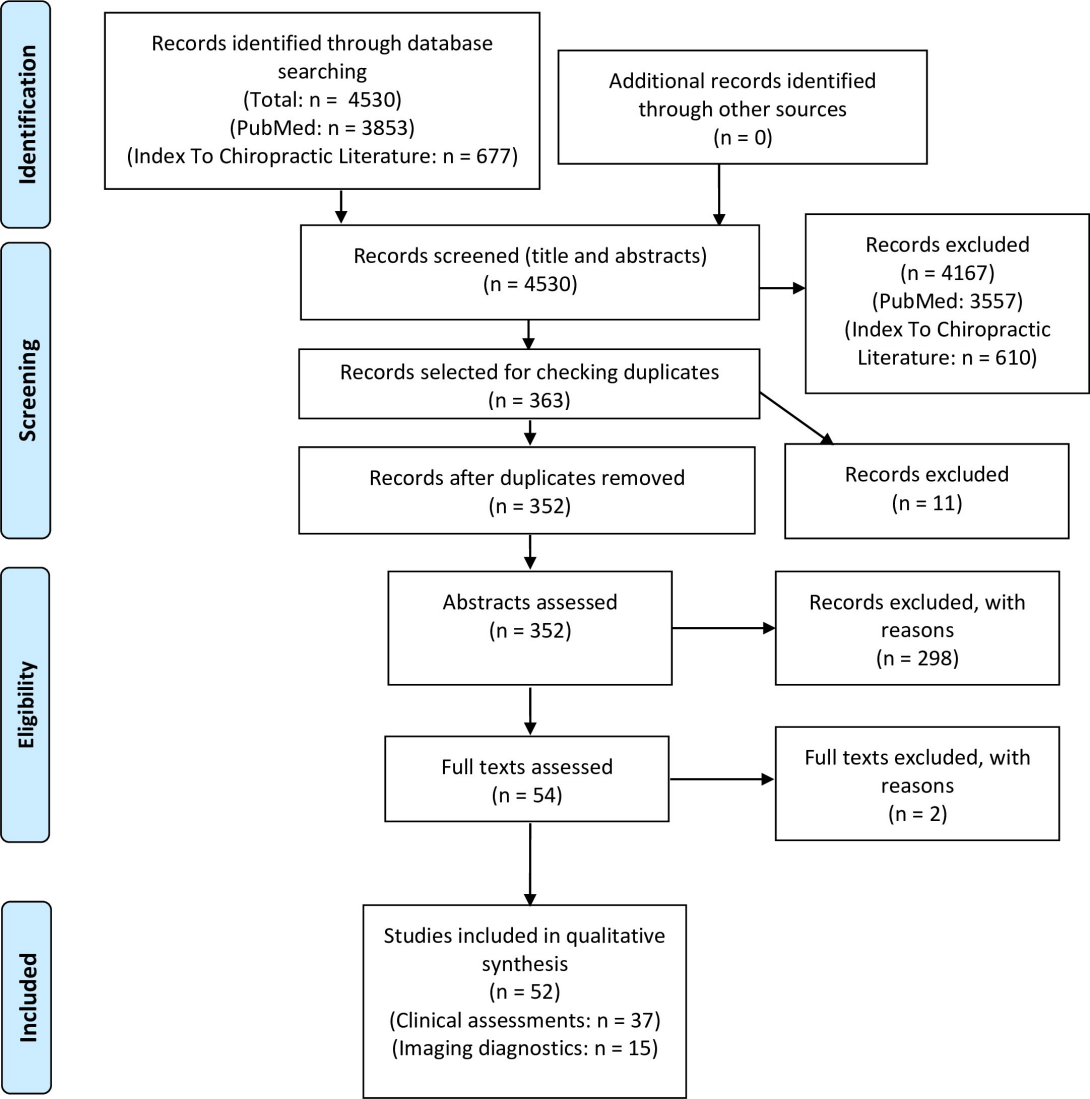
Flow diagram demonstrating the flow of studies through the review.

A systematic review was conducted following the recommendations of Preferred Reporting Items for Systematic reviews and Meta-Analyses (PRISMA) [13]. Within the scope of a master’s thesis, the databases Medline (PubMed) and Index to Chiropractic Literature were systematically searched until November 2017. Therefore, the present review was not prospectively registered. In order to update the search for the present publication, the search strategy was upgraded and repeated until October 15, 2020. In PubMed the following search terms were combined using the Boolean Operator “OR”: “leg length discrepancy, leg length inequality, limb length discrepancy, limb length inequality, long limb and long foot” and limited to humans. Then, the search terms “physical therapy, physiotherapy, orthopaedic, orthopaedics, orthopedic, osteopathy, chiropractic, alternative medicine, podiatry and podiatric”were combined using the Boolean Operator “OR” and limited to humans. Additionally, the search terms “measurement, measure, assessment, test, physical exam, physical examination, clinical examination, imaging diagnostics and diagnostic imaging”were combined and limited using the same procedure. For the updated search we added the following search terms and combined them with the operator “OR” and limited to humans: reliability, reproducibility, validity, accuracy, comparability, comparison, variability, variation, variance. Finally, all combined search combinations using the operator “OR” were then combined using the Boolean Operator „AND“. In Index to Chiropractic Literature the search term “leg length” was combined with the search terms “leg-length”, “limb length” and “limb-length using the operator “OR”. Furthermore, the reference lists of all eligible diagnostic studies and reviews, as well as the reference lists of reviews with similar topics were screened. A flow diagram of searches for studies is presented in **Fig 1**.

### Eligibility of studies

Full text studies regarding clinical assessments and imaging diagnostics for the diagnosis of LLD, which reported the clinimetric properties, involving validity or accuracy, and reliability for the assessment of LLD or leg lengths, were included. Further inclusion criteria were English or German language, for studies dealing with clinical assessments a year of publication ≥1983 and for studies regarding imaging diagnostics a year of publication ≥2000 to provide a review of updated literature.

### Exclusion criteria

All studies that did not aim at examining LLD or leg lengths in humans and did not meet the aforementioned inclusion criteria were excluded.

### Selection of studies and analysis

Titles and abstracts of the identified studies were screened independently by two reviewers (P.F. and M.A.). Full texts were reviewed by the two reviewers independently. Any disagreements were discussed between reviewers and clarified. A third author (A.K.) was consulted if agreement of full-text inclusion could not be reached. In that case, the third author served as arbiter for final decision about inclusion or exclusion of the respective study. Additional hand search of literature was conducted continuously by the authors for any further manuscripts dealing with LLD.

### Quality assessment of included studies

**Fig 2:**
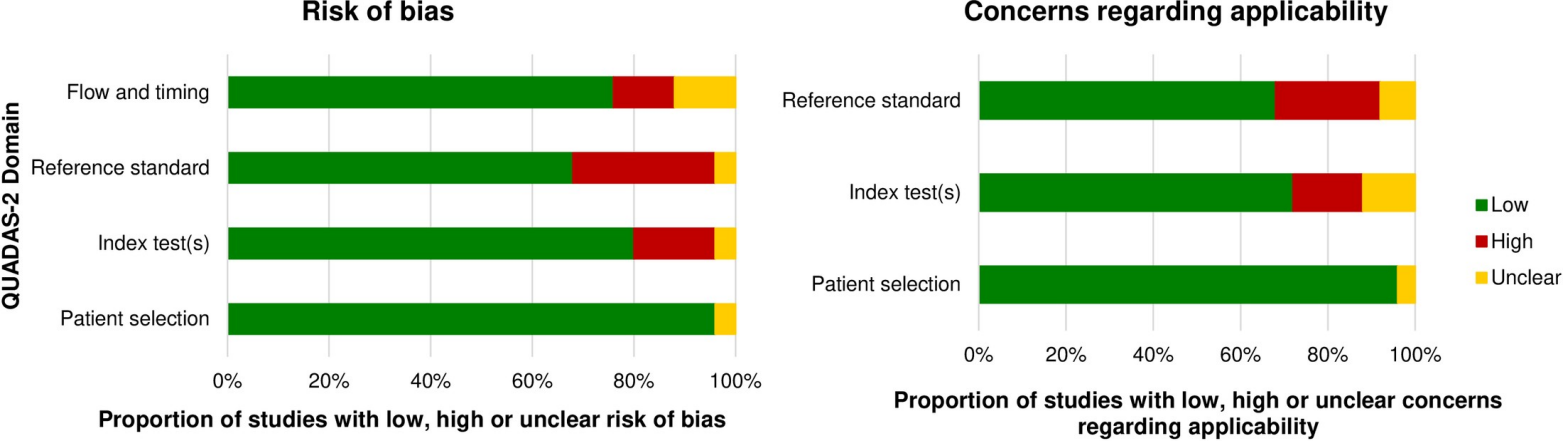
Proportion of studies on clinical assessments with low, high or unclear risk of bias and concerns regarding applicability assessed using the QUADAS-2 tool.

**Fig 3:**
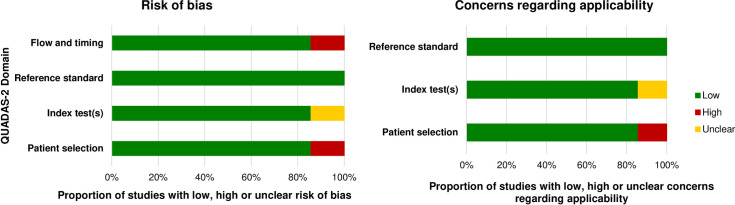
Proportion of studies on imaging diagnostics with low, high or unclear risk of bias and concerns regarding applicability assessed using the QUADAS-2 tool.

The revised “Quality Assessment of Studies of Diagnostic Accuracy (QUADAS-2)” tool was completed to evaluate the risk of bias of the included diagnostic studies and studies investigating concurrent validity [14] by two authors (P.F.; M.A.) independently (**Tables 1 and 2; Figs 2 and 3**). This tool involves four domains: patient selection, index test, reference standard, and flow and timing. Each domain is evaluated in the form of risk of bias. Furthermore, a statement on concerns regarding applicability is provided for the first three domains. Signalling questions are incorporated to assist judge risk of bias. The included studies were categorized as having a “high,” “low,” or “unclear” overall risk of bias or concerns regarding applicability.

**Table 1:** QUADAS 2- tool for the evaluation of quality of the included validity studies on clinical assessments for the measurement of leg length discrepancy (n = 25).

	Risk of Bias				Applicability Concerns		
Study	Patient Selection	Index Test	Reference Standard	Flow and Timing	Patient Selection	Index Test	Reference Standard
Aguilar et al. (2017)	L	H	H	H	L	H	H
Aspegren et al. (1987)	U	H	H	U	U	H	H
Badii et al. (2014)	L	L	L	L	L	L	L
Beattie et al. (1990)	L	L	L	L	L	L	L
Betsch et al. (2019)	L	L	U	L	L	L	U
Cleveland et al. (1988)	L	L	L	L	L	L	L
Cooperstein et al. (2003)	L	L	H	H	L	L	H
Cooperstein et al. (2004)	L	L	H	H	L	L	H
Edeen et al. (1995)	L	L	L	L	L	L	L
Farella et al. (2005)	L	H	H	L	L	H	H
Friberg et al. (1988)	L	L	L	L	L	L	L
Gross et al. (1998)	L	L	L	L	L	L	L
Hanada et al. (2001)	L	L	L	L	L	L	L
Harris et al. (2005)	L	L	L	U	L	U	L
Jamaluddin et al. (2011)	L	L	L	U	L	L	L
Krettek et al. (1996)	L	L	L	L	L	L	L
Lampe et al. (1996)	L	L	L	L	L	L	L
Montgomery et al. (1995)	L	L	L	L	L	U	L
Neelly et al. (2013)	L	L	L	L	L	L	L
Petrone et al. (2003)	L	L	L	L	L	L	L
Piyakunmala & Sangkomkamhang (2018)	L	U	L	L	L	U	L
Rhodes et al. (1995a)	L	H	H	L	L	H	H
Rhodes et al. (1995b)	L	L	L	L	L	L	L
Sayed-Noor et al. (2009)	L	L	H	L	L	L	U
Woerman & Binder-MacLeod (1984)	L	L	L	L	L	L	L

L = Low; H = High; U = Unclear.

**Table 2:** QUADAS 2- tool for the evaluation of quality of the included validity studies on imaging diagnostics for the measurement of leg length discrepancy (n = 7).

	Risk of Bias				Applicability Concerns		
Study	Patient Selection	Index Test	Reference Standard	Flow and Timing	Patient Selection	Index Test	Reference Standard
Jensen et al. (2017)	L	L	L	L	L	L	L
Khakaria et al. (2011)	L	L	L	L	L	L	L
Kjellberg et al. (2012)	L	L	L	L	L	L	L
Rannisto et al. (2011)	H	U	L	H	H	U	L
Reina-Bueno et al. (2017)	L	L	L	L	L	L	L
Sabharwal et al. (2006)	L	L	L	L	L	L	L
Tipton et al. (2016)	L	L	L	L	L	L	L

L = Low; H = High; U = Unclear.

**Fig 4:**
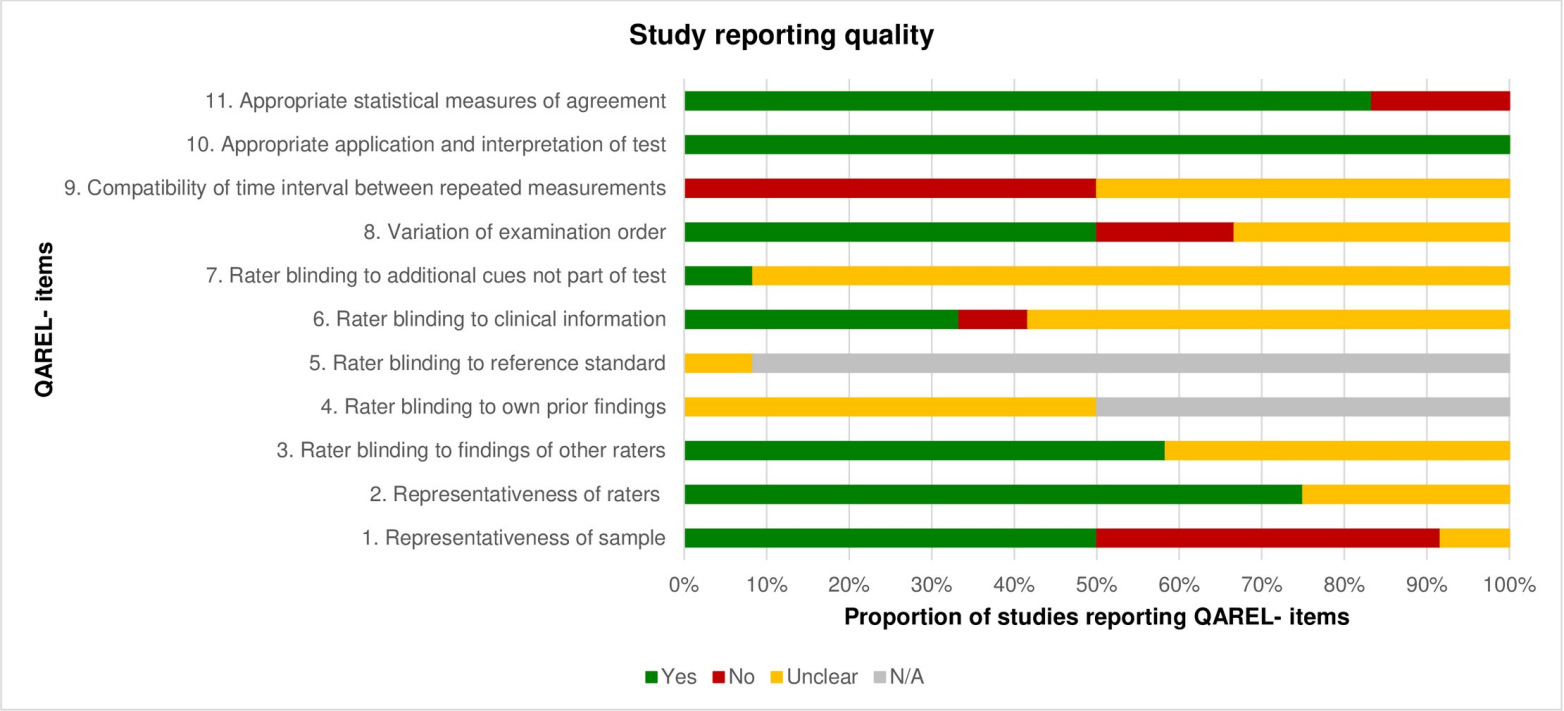
Assessment of study reporting quality of the included reliability studies on clinical assessments using the QAREL- checklist. N/A = not applicable.

**Fig 5:**
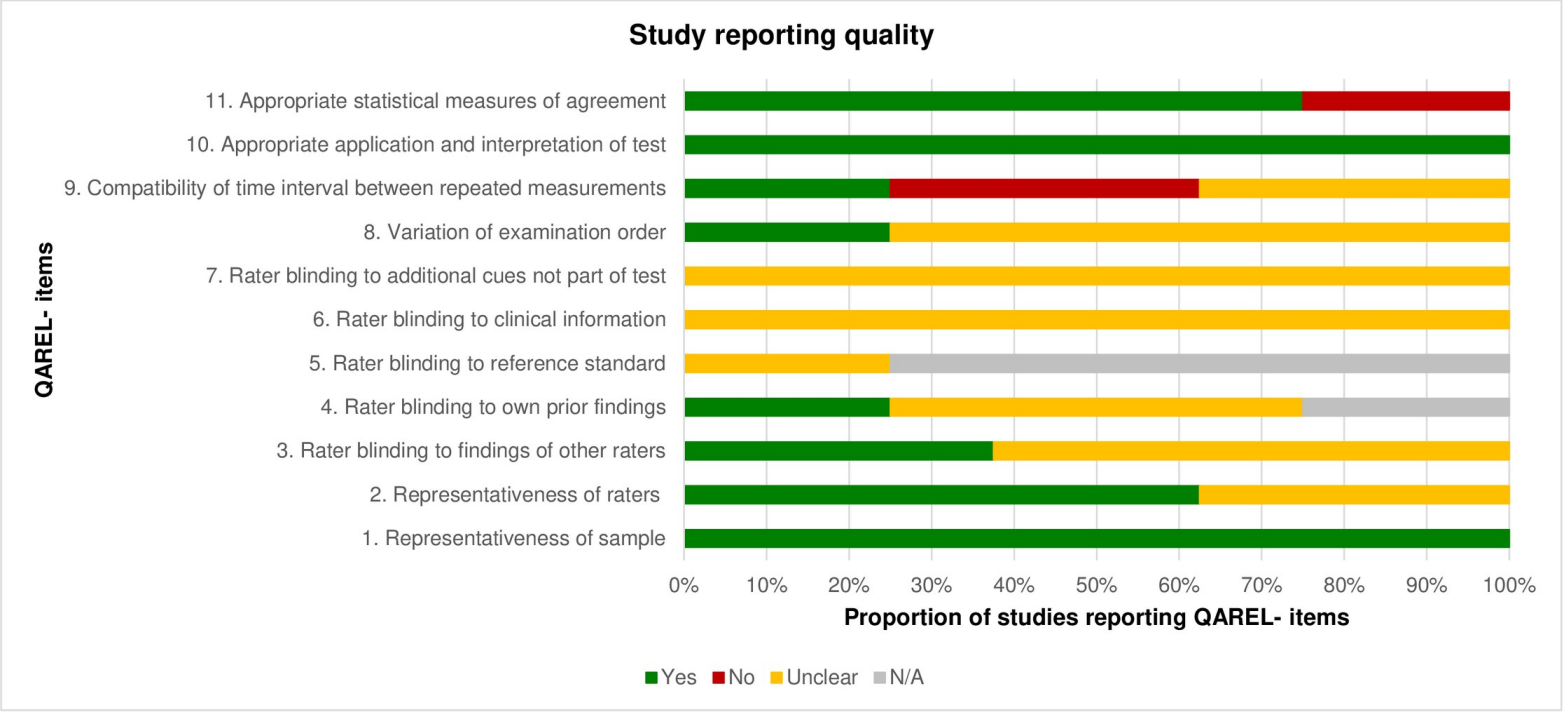
Assessment of study reporting quality of the included reliability studies on imaging diagnostics using the QAREL- checklist. N/A = not applicable.

For analysis of risk of bias of studies investigating reliability the “Quality Appraisal of Diagnostic Reliability (QAREL)” tool [15] was used (**Tables 3 and 4; Figs 4 and 5**). The tool incorporates 11 items, asking about e.g. representativeness of the sample, blinding of raters, time interval between measurements, application and interpretation of the measurements, statistical methods of agreement, etc. Each item was judged in terms of its significance for the quality of the study [16] by two authors (P.F.; M.A.) independently. If an item was judged with “yes” it was coded with “1”, resulting in a maximal achievable value of “11”. In the present study a value ≤ 4 represented a high, a value ≥ 5 to 7 a moderate and a value ≥ 8 a low risk of bias [17]. Any disagreements were discussed between reviewers and clarified. A third author (A.K.) was asked if agreement of evaluation could not be reached.

**Table 3:** QAREL-checklist for the evaluation of quality of the included reliability studies on clinical assessments for the measurement of leg length discrepancy (n = 12).

Study	Items												Risk of bias (Quality)
	1	2	3	4	5	6	7	8	9	10	11	Sum	
Cooperstein et al. (2017)	N	U	U	U	N/A	U	U	N	N	Y	Y	2	High (Low)
Cooperstein & Lucente (2017)	Y	Y	Y	U	N/A	Y	Y	Y	N	Y	Y	8	Low (High)
De Boer et al. (1983)	N	Y	Y	U	N/A	Y	U	Y	N	Y	Y	6	Moderate (Moderate)
Hellsing (1988)	Y	U	U	U	N/A	U	U	U	N	Y	Y	3	High (Low)
Holt et al. (2009)	Y	Y	Y	N/A	N/A	U	U	Y	U	Y	Y	6	Moderate (Moderate)
Junk et al. (1992)	N	U	U	N/A	N/A	U	U	U	U	Y	N	1	High (Low)
Nguyen et al. (1999)	Y	Y	Y	N/A	N/A	U	U	Y	N	Y	Y	6	Moderate (Moderate)
Schneider et al. (2007)	Y	Y	Y	N/A	N/A	Y	U	U	U	Y	Y	6	Moderate (Moderate)
Schwartzbauer & Hart (2011)	N	Y	Y	N/A	N/A	U	U	N	U	Y	Y	4	High (Low)
Shambaugh et al. (1998)	U	Y	U	U	N/A	U	U	U	U	Y	N	2	High (Low)
Terry et al. (2005)	Y	Y	Y	U	U	Y	U	Y	N	Y	Y	7	Moderate (Moderate)
Woodfield et al. (2011)	N	Y	U	N/A	N/A	N	U	Y	U	Y	Y	4	High (Low)

N = No; Y = Yes; U = Unclear; N/A = Not applicable.

**Table 4:** QAREL-checklist for the evaluation of quality of the included reliability studies on imaging diagnostics for the measurement of leg length discrepancy (n = 8).

Study	Items												Risk of bias (Quality)
	1	2	3	4	5	6	7	8	9	10	11	Sum	
Clavé et al. (2018)	Y	U	U	U	N/A	U	U	U	N	Y	Y	3	High (Low)
Guggenberger et al. (2014)	Y	Y	U	N/A	U	U	U	U	U	Y	Y	4	High (Low)
Konermann & Gruber (2002)	Y	U	Y	U	N/A	U	U	U	U	Y	N	3	High (Low)
Lazennec et al. (2016)	Y	U	U	N/A	N/A	U	U	U	U	Y	Y	3	High (Low)
Meermanns et al. (2011)	Y	Y	U	U	U	U	U	U	Y	Y	N	4	High (Low)
Poutawera & Stott (2010)	Y	Y	U	Y	N/A	U	U	Y	N	Y	Y	6	Moderate (Moderate)
Riad et al. (2010)	Y	Y	Y	Y	N/A	U	U	Y	Y	Y	Y	8	Low (High)
Sabharwal et al. (2007)	Y	Y	Y	U	N/A	U	U	U	N	Y	Y	5	Moderate (Moderate)

N = No; Y = Yes; U = Unclear; N/A = Not applicable.

## Results

### Flow of studies through the review

The updated search revealed 3853 studies in PubMed and 677 studies in Index to Chiropractic Literature (**Fig 1**). After titles and abstracts were screened 296 studies were selected in PubMed and 67 studies in Index to Chiropractic Literature. The citations for the selected publications (n = 363) were imported into Endnote software, and duplicates (n = 11) were filtered automatically. After duplicates were removed (n = 11), abstracts of the remaining studies (n = 352) were assessed in depth. After that, 298 articles were excluded. Full texts of the remaining 54 articles were checked for eligibility criteria. Two articles [18, 19] were excluded for the following reasons: measurement of leg length using composite femurs [18]; correlation between anatomical leg length and perception of leg length discrepancy [19]. Of the remaining 52 articles, 37 articles on clinical assessments [20–56] and 15 articles on imaging diagnostics [57–71] were finally included and analyzed.

### Quality of the included studies

Characteristics and main results of the included studies on clinical assessments and imaging diagnostics are provided in **S1 and S2 Tables**. Thirteen studies on the validity of clinical assessments [22, 23, 25, 31, 33–35, 41, 42, 44, 46, 48, 55] had a low risk of bias and low concerns regarding applicability for all domains using QUADAS-2 tool (**Table 1**). One study on the reliability of clinical assessments [27] had low risk of bias using the QAREL- checklist (**Table 3**). Six studies on the validity of imaging diagnostics [59–61, 67, 70, 71] had a low risk of bias and low concerns regarding applicability for all domains using QUADAS-2 tool (**Table 2**). Only one study on the reliability of imaging diagnostics [68] had low risk of bias using the QAREL- checklist (**Table 4**).

### Data synthesis

A quantitative data synthesis with analyses of sensitivity and specificity was not performed because of heterogeneity and nonuniformity of data across included studies. Eight relevant validity studies on clinical assessments [20, 21, 24, 28, 29, 32, 49, 50] did not involve an adequate reference standard, needed to determine sensitivity and specificity [72]. Therefore, the results were summarized in a descriptive way. Validity and/or reliability of clinical assessments and imaging diagnostics were assessed in the included studies; results are reported in **S1 and S2 Tables**.

### Clinical assessments

#### Tape measure method

Sixteen studies reported on the determination of validity and/or reliability and/or accuracy of the tape measure method for measuring LLD [20, 22–25, 31, 33, 36, 39–42, 44, 50, 54, 55] (**S1 Table**). Further terms used were variability, variance or variation. The tape measure method was described as following: 1.) measurement from spina iliaca anterior superior (SIAS) to the medial malleolus [20, 23–25, 31, 33, 36, 39, 40, 42, 44, 50, 54, 55]; 2.) measurement of the distance between the SIAS and the lateral malleolus [20, 22, 41, 55]; 3.) measurement from SIAS to navel [55]; 4.) measurement from SIAS to xiphoid [55]; 5.) measurement from os pubis to malleolus medialis [31].

In four studies on tape measurement from spina iliaca anterior superior (SIAS) to the medial malleolus the reference standard CT-scanogram was used [23, 36, 39, 44], in one study the slit scanogram radiographic measurement [54], in one study the split scanogram including the hip, knee and ankle [55]. In eight studies [23–25, 31, 33, 42, 50, 55] different radiographic measurement methods served as the reference standard. In one study [40], an ultrasound measurement was performed, however, it appeared to be another index test rather than a reference standard. In one study on tape measurement from SIAS to the lateral malleolus, the radiography of the pelvis was used as reference test [22], in one study the slit scanogram radiographic measurement [54], in another study the split scanogram including the hip, knee and ankle [55], and in one study the ultrasound and teleroentgenography [41]. The tape measure methods from SIAS to the navel and from SIAS to the xiphoid were compared with the split scanogram including hip, knee and ankle as being the reference standard in one study [55]. In the study using the tape measure method with the landmarks os pubis and malleolus medialis the orthoroentgenography and the pelvic radiography were carried out as reference standards [31]. In one study no reference standard was used [20]. Only in a few studies [36, 39, 44, 54] it was reported, that the tape measure method is reliable and/or valid. In the most studies [22–25, 31, 33, 41, 42, 50, 55] it was concluded, that tape measurement methods were less accurate, revealed wide variation, weak correlation with other methods and disagreement with radiography, lead to miscalculation of small leg length discrepancy, and should be used with caution. There is high evidence that the tape measure method is less valid and reliable.

#### Block test

Validity and/or accuracy and/or reliability of the block test or standardized wooden boards was determined in eleven studies [21, 22, 31, 33, 36, 40–42, 47, 54, 55] (**S1 Table**). The reference standard was the radiography/roentgenography in eight studies [21, 22, 31, 33, 41, 42, 47, 55], the slit scanogram radiographic measurement in one study [54], the split scanogram including the hip, knee and ankle in one study [55], and the CT-scanogram in one study [36]. In another study, an ultrasound technique and a tape measure method was used, however, it appeared that all measurements were used as index tests rather than reference standard [40]. The block test was considered reliable, accurate and relevant or being superior to tape measure method in five studies [22, 36, 42, 54, 55]. However, poor validity and reliability compared with orthoroentgenography was reported in two studies [31, 47]. Furthermore, it was considered less accurate than ultrasound measurement [41] and radiographic measurement [33]. Two studies seemed methodologically insufficient [21, 40] so that a clear conclusion on accuracy, validity or reliability could not be provided based in their results. Overall, study results differed, so that there is moderate evidence that the block test is or is not valid and reliable for determining LLD.

#### “Iliac crest palpation and book correction” method

The reliability and validity of the “iliac crest palpation and book correction” method (ICPBC) was evaluated in one study [35] (**S1 Table**). Here, the standing radiographic measurement served as reference standard. Because of high reliability and fair validity of the ICPBC method, it was recommended to use iliac crest palpation to identify LLD when there is no history of pelvic deformity and the iliac crests can be simply palpated. Based on only one high quality study with low risk of bias, the evidence that the ICPBC method is reliable and fairly valid is strong, however, further studies are needed to confirm this conclusion.

#### Prone leg check methods

In twelve identified publications [26–30, 38, 45, 48, 49, 51–53], reliability and/or validity of the measurement of leg lengths with the participant in prone position was examined (**S1 Table**). Thereby, the following methods were described: 1.) measurement of the difference between the posterior aspects of the soles using a millimeter ruler [48, 49]; 2.) activator method, i.e. applying pressure in cranial direction through the long axis of the legs using the thumbs and determining whether the leg lengths were equal or whether one leg was shorter compared to the other [45]; 3.) instrumented compressive leg check, i.e. the participants wore a modified surgical boot with a screw mounted at the medial aspects of the wooden left and right soles. During each measurement left or right shoe height was randomly increased using a maximum of 6 shims (0–6; 6 = 9.6 mm) to artificially induce LLD. The difference between the right and left leg length, that was displayed by the screws, was determined using a ruler that was placed at the end of the examination table between the participant’s feet [28, 29]. The method was also used in a further study, however, without placing shims beneath the shoe sole [26]; 4.) assessment of leg length discrepancy in prone position by visually comparing the level of the medial malleoli [27]; 5.) determination of LLD with the participant in prone position with the knees extended and then flexed to 90° [30, 38, 51, 52]; 6.) determination of LLD with the participant in prone position with the knees extended and left head rotation [52]; 7.) measurement of LLD with the participant in prone position and the hip in extension [52]; 8.) Derifield-Thompson leg check [53].

A reference standard (standard radiography) was reported only in the studies by Rhodes et al. [48, 49]. The assessment of leg length discrepancy in prone position was predominantly considered reproducible and accurate [26, 28, 29, 38, 45, 49, 51, 53]. In a few studies, reliability was reported poor to fair [27, 30, 52]. The studies investigating validity of the prone leg check showed contradictory results, with one reporting good validity [48], and the other reporting poor validity [49]. There is low evidence that prone leg check methods are or are not valid and reliable for determination of LLD. Further high quality studies are needed to draw a valid conclusion.

#### Hand-held devices

Three studies were identified, where a hand-held device was used for the measurement of LLD [20, 34, 46]. Gross et al. [34] investigated the reliability and validity of using a calibrated pelvic leveling device (**S1 Table**). The reference standard was a radiographic measurement of the lower extremity. Petrone et al. [46] evaluated the reliability and validity of measuring the difference of iliac crest height (mm) using the PALM (**S1 Table**). The reference test was a pelvic radiography. Aguilar et al. [20] investigated the relationship between the method measuring the distance between malleolus medialis (DIMG) and lateralis (DEMG) and the ground and other clinical measurements, including the PALM and the pelvimeter. A reference standard was not used. Good reliability and moderate validity for the pelvic leveling device and the PALM were concluded in two studies [34, 46], however, in one of those studies [34] it was pointed out, that the results of reliability and validity do not support clinical use of the device to determine LLD, whereas the device was considered an alternative to radiographic measurement in the other study [46]. The third study [20] did not provide beneficial information on reliability and validity of the measurement of leg length discrepancy using the PALM and pelvimeter. Based on two useful high quality studies with low risk of bias, the evidence that hand-held devices for detecting LLD are moderately valid and reliable is strong. However, further high quality studies are needed to confirm this conclusion.

#### Distance between the malleolus lateralis or medialis and the floor

One study using the distance between the malleolus lateralis or medialis and the floor to determine LLD was identified [20]. Here, the relationship using the calculation of correlations between this index test and further clinical assessments measuring LLD in consideration of foot posture was investigated (**S1 Table**). Further methods were: 1.) tape measure method using the distance between anterior spina iliaca superior and malleolus medialis; 2.) tape measure method using the distance between anterior spina iliaca superior (ASIS) and malleolus lateralis; 3.) measurement of the difference between the left and right pelvic crest using a pelvimeter; 4.) measurement of the asymmetry between the left and right ASIS using a palpation meter. The authors concluded, that the index test was related to other methods to determine leg length asymmetry. However, they further recommended not to use the measurements with the malleolus lateralis and medialis alternatively, because they significantly differed. An appropriate reference standard was not included. There is low evidence that measuring the distance between the malleoli and the floor is a valid method for assessing LLD.

#### Supine leg check method

Six studies were found, in which reliability of LLD measurements with the participants in supine position was investigated [27, 31, 32, 49, 52, 56] (**S1 Table**). Validity was determined in three of those studies [31, 32, 49]. In one reliability study, the leg check was considered reliable [56]. In another reliability study, no agreement between the prone and supine leg check was concluded [27]. In the last reliability study, poor agreement was found for the supine leg check between examiners [52]. In the studies evaluating validity and reliability, poor validity of the supine leg check method was concluded [32, 49], which was also confirmed by Edeen et al. [31]. In one of those studies, poor reliability was found [32], in the other study reliability was reported excellent [49]. A reference standard was only included in two studies evaluating validity [31, 49]. Overall, there is low to moderate evidence that the supine leg check method is or is not valid and reliable for determinig LLD. Further high quality studies are needed to draw a valid conclusion.

#### Palpation and visual assessment

Agreement of the palpation of the unilateral sacral prominence and radiography (reference standard) to determine LLD was investigated by Montgomery et al. [43] (**S1 Table**). Leg length was measured bilaterally from the upper edge of the acetabulum to the lower edge of the calcaneus. During the clinical measurement patients were positioned in prone and the assessor palpated the inferior angle of the sacrum, in order to identify unilateral sacral prominence. It was concluded that the palpation of the unilateral sacral prominence was as accurate as radiography for determination of an anatomical short leg under 9 mm. One study was identified using a visual assessment of leg length discrepancy during palpation of iliac spines [37], revealing some agreement between the results of three measurements. Based on only one study with acceptable quality, no valid conclusion on the evidence on validity and reliability of this method can be drawn.

### Imaging diagnostics

#### Bi-planar imaging system EOS^TM^

Three studies were included that used the bi-planar imaging system EOS^TM^ for the determination of LLD [57, 59, 63]. In one study [59], preview images [radiation dose = 2.99 Gray x square centimeter (mGy x cm^2^)] recorded by the system were compared with diagnostic images (radiation dose = 239.26 mGy x cm^2^). Furthermore, reliability of the methods was determined (**S2 Table**). No significant difference was found between methods. The authors concluded that preview images are comparable to diagnostic images and could be used for follow-up examination to reduce radiation exposure. In the other studies [57, 63], 2-D- [63] and 3D- measurements [57, 63] using the EOS^TM^- system were considered accurate and highly reliable. However, both studies showed methodological concerns. A reference standard was included in none of those studies. Based on only one high quality study with low risk of bias, and two low quality studies with high risk of bias, the evidence that the EOS^TM^- system is valid and reliable is low.

#### Full-length standing anteroposterior radiography

Four studies on the measurement of LLD using full-length standing anteroposterior radiography as index test were included [58, 67, 69, 70]. Here, high to almost perfect reliability of the methods was determined (**S2 Table**). Two studies investigated validity appropriately [67, 70]. The full-length standing radiography was considered valid. In one study it was concluded that full length standing anteroposterior radiography is superior to scanogram (reference standard) for the measurement of LLD [70]. Moreover, the authors recommended to commonly use the standing radiograph for assessing LLD in patients with angular deformities of the lower extremity, because it is similar to the measurement using the scanogram with a lower radiation dose. In summary, there is high quality evidence that full-length standing anteroposterior radiography is a valid method for determination of LLD, however, evidence regarding reliability is low to moderate.

#### Scanogram

One study was identified, where the reliability of LLD- measurements using CT- scanogram was investigated [65]. Although agreement of repeated measurements was almost perfect, CT- scanograms should be carried out more than once and should be double-checked by the surgeon. No reference standard was used. There is moderate evidence, that the scanogram is reliable for measuring LLD. No conclusion on validity can be drawn. High quality studies investigating validity of the scanogram are needed.

#### Pelvic radiography with pelvic landmarks

Two studies were identified where reliability and validity of pelvic radiography with pelvic landmarks was assessed [61, 71] (**S2 Table**). The method was compared with full-length standing radiograph [71] or CT-scanogram [61] (reference standards). The authors concluded that the use of pelvic radiography with landmarks should be considered cautious when determining LLD because of the limiting comparability to full-length standing radiography [71] and CT-scanogram [61]. A further study was found, where only reliability of the pelvic radiography for the measurement of LLD was assessed [64] (**S2 Table**). The method using the pelvic reference interteardrop line and center of the femoral head were considered the most accurate methods for the preoperative assessment compared to the measurement of true LLD using full-leg radiography. However, correlation or agreement between the methods were not determined, limiting this conclusion. There is strong evidence, that validity of the pelvic radiography with pelvic landmarks is low. There is low evidence that the method is reliable.

#### Picture archiving and communication system (PACS)

One study was found that explored the comparability of accuracy and reproducibility of LLD measurement between PACS and a standard hard-copy radiograph [60]. Measurements were conducted by two raters independently. A comparable reliability was declared for both methods and an excellent agreement was found between both methods. Therefore, a change from hard-copy film to PACS was recommended (**S2 Table**). Based on one high quality study with low risk of bias, there is high evidence, that measurement of LLD using PACS is valid and reliable. However, further high quality studies are needed to substantiate this conclusion.

#### Ultrasound methods

One study, where the authors investigated the reliability of LLD measurement using the laser-based ultrasound method was found [66]. The ultrasound scanning head of the ultrasound apparatus was fixed at a rod and positioned perpendicular to the tissue interface of the anterior hip region. The distance between the highest point of the femoral head and the floor was measured by two blinded physiotherapists at two consecutive days. Reference standard was the radiographic measurement. The authors concluded an excellent reliability of ultrasound and agreement with radiographic measurement (**S2 Table**). Therefore, it could be an alternative to the radiographic measurement of LLD, because it is practicable and non-invasive. Another study investigating the reliability of the sonographic measurement of LLD was identified [62]. A good reproducibility of the determination of total leg length, length of the femur and length of the tibia was concluded, however, the calculation of coefficients of correlation or agreement and the comparison to a reference standard lacked (**S2 Table**). Based on low quality studies with high risk of bias, there is low evidence, that ultrasound is a valid and reliable method to determine LLD.

#### Magnetic resonance imaging

One study was found where LLD was investigated using magnetic resonance imaging (MRI) [68] (**S2 Table**). Based on sagittal T1-weigthed MRI images of the lower extremity, the length of the pelvis, femur, tibia and calcaneus was measured with the patients positioned in supine and the legs fully extended. The measurements were conducted by two experienced examiners and were repeated after two weeks. Excellent reliability was reported. There is high quality evidence, that magnetic resonance imaging is reliable for assessing LLD. However, high quality studies on the validity of magnetic resonance imaging are needed.

## Discussion

Leg length discrepancy is a common finding in the human population. Numerous methods are used in clinical practice. To the best of the authors’ knowledge, this is the first systematic review summarizing study results on the validity and reliability of clinical assessments and imaging diagnostics for the measurement of LLD used in the clinical arena. The main finding was, that the use of almost all clinical assessments is questionable, because of strong evidence that the methods are not valid, reliable and accurate, i.e. tape measure method, or because of little evidence to draw any conclusion. Based on the results of the present review, only the block test can be recommended. Strong evidence for usage was only found for the full-length standing anteroposterior radiography, however, high radiation, high costs, low practicability, and limitations for determination of functional leg length discrepancy, may restrict its use in clinical practice. The use of the block test as well as full-length standing anteroposterior radiography for determination of LLD was also recommended by experienced orthopedists in a recent narrative review [73]. Following, the implication of especially these methods are discussed.

### Clinical assessments—Implication for clinical practice

The block test, which is considered the „indirect”method for measuring LLD [12], showed a higher reliability, sensitivity (55%) and specificity (89%) compared to using a tape measure (sensitivity = 45%; specificity = 56%) [22], which is considered the “direct” clinical measure method [12]. Both clinical assessments were described more reliable and relevant than CT-scanogram measurements for determination of LLD in patients after femoral fracture [36]. In only one out of 20 cases the block test identified the wrong leg as being shorter compared to pelvic radiography [22]. In contrast, other study results suggested the block test being not as accurate as imaging diagnostics and therefore, it should be used for screening of patients under suspicion of having LLD [12]. Furthermore, high values of interobserver variance (mean difference: 1.01 cm; 95% CI: 2.2 cm) led to the conclusion that the block test is unacceptable for clinical decision making [54]. It has to be considered, that contradictory results may have been caused by different ages of participants in the studies (23–85 years [22] versus 1.5–19 years [54]), respectively. Overall, sample sizes differed between studies of clinical assessments and may have influenced statistical analyses and interpretations.

The most included studies on the block test had an acceptable or even high quality, except for the studies by Aspegren et al. [21] and Junk et al. [40], because they showed high risk of bias. Therefore, the block test seems to be beneficial, as it is completed with the participant in standing position and the difference of functional as well as anatomical leg length is captured simultaneously. However, palpation and visual analysis during measurement may represent potential sources of bias.

Devices, such as the palpation meter or the pelvic leveling device demonstrated good reliability [34, 46], however, for the latter, validity was only moderate as compared with imaging diagnostics (ICC = 0.64) [34]. In contrast to the pelvic leveling device, the determination of LLD using the PALM was considered an alternative to radiographic measurement. Therefore, using devices for assessing LLD may be an additional option in the clinical arena.

### Imaging diagnostics—Implication for clinical practice

Sabharwal & Kumar [12] described that the ideal diagnostic imaging procedure to measure LLD would be accurate, reliable, readily available and affordable. Moreover, it should allow for visualization of the complete lower extremity, should use a low radiation dose and have no magnification error. Using the teleoroentgenogram was considered the first choice for initial examination of patients with LLD [12] for a couple of reasons. The average magnification of the teleoroentgenogram compared to the scanogram was 4.6% [70]. An excellent correlation between measurements of the teleoroentgenogram and CT- scanogram was found. Intra- and interobserver- reliability of measurements of LLD using the teleoroentgenogram were demonstrated [69]. The CT- scanogram provided reliable measurements of LLD using minimal magnification as well [12]. However, radiation exposure of the scanogram was 1.6 to 3.8 times increased compared to the teleoroentgenogram [70].

The PACS may represent a reliable alternative to hardcopy radiography [60]. Although the respective study was rated being at low risk of bias and low concerns of applicability, the conclusion should be considered with caution, because data of standing limb images were analyzed retrospectively, which did not allow for analyzing possible confounding factors that may have been associated with the measurements [74].

Magnetic resonance imaging noninvasively produces high-resolution anatomy images and is useful for the diagnosis of various musculoskeletal conditions [75]. An excellent intra- and interobserver- reliability was observed for examination of LLD [68]. However, a reference standard lacked, so that no information about validity of results was available. Therefore, a comparison of LLD measurements using MRI with the gold standard, which is considered the radiographic scanogram, would be needed [12, 76]. An advantage of MRI is that it does not administer radiation to the patient, however, it may be more expensive, may require sedation in some patients, often needs a longer time to schedule and to carry out the examination, and may be not allowed in patients with specific implanted devices [12].

### Limitations

An analysis of sensitivity and specificity was only performed in four studies [22, 32, 41, 47] with one based on an insufficient reference standard [32]. Furthermore, eight studies on clinical assessments did not include a (valid) reference standard [20, 21, 24, 28, 29, 32, 49, 50]. As the reference standard is a decisive determinant of diagnostic accuracy of a test [77], the studies without a reference standard may not be considered a diagnostic accuracy study (https://www.equator-network.org/reporting-guidelines/stard/) and the intended use of QUADAS-2 quality assessment may have been limited. A transient solution for the analysis of studies without a reference standard may be using two critical appraisal tools [78]. Schröder et al. [78] evaluated the methodological quality of the reproducibility studies by assessing reliability using the reliability box of the Consensus-based Standards for the selection of health status Measurement Instruments (COSMIN) and the validity studies including a reference standard using QUADAS-2. In the present study, the QAREL- tool was used as reported previously [79], because this tool was developed according to QUADAS-2 [14, 77]. Another challenge is the comparison of accuracy of multiple tests to assess the target condition compared to a reference standard (comparative study) and the evaluation of accuracy of a single-test to assess the target condition compared to a reference standard (single-test or non-comparative studies) [72]. A clinically relevant comparative question can result in a mix of single-test (non-comparative) studies and comparative studies and it is up to the review author how to tailor the QUADAS-2 quality assessment for the comparative question. This problem needs new statistical approaches that are under development. In the present study, a complete discrimination of methodological approaches in the analysis of the included studies, such as different study designs, populations, statistical analyses, number and variation of index tests, or use of reference standard could not be met. Furthermore, many terms of measurement quality criteria were used in the included studies. Thereby, it has to be considered, that there are fundamental differences between the concepts of reliability and agreement [80], which may not have been considered comprehensively among analysis of all studies. Therefore, the results of this systematic review on clinical assessments and imaging diagnostics to determine LLD should be considered as preliminary results, that need to be further investigated.

## Conclusion

The block test appears to be the most useful clinical assessment to measure LLD, followed by using devices, such as the pelvimeter. All other clinical tests seem to be not useful and may therefore be avoided by the clinicians and physiotherapists. Full-length standing anteroposterior radiography may be used as global reference standard to measure the anatomic LLD when comparing clinical methods and imaging diagnostics. The alternative measurements PACS and MRI need to be further investigated. The findings of the review may help clinicians to select the most valid, reliable and accurate clinical assessment to determine LLD in their patients and to make a decision, whether a compensation for LLD is indicated or not.

## Supporting information

S1 Checklist PRISMA 2009 checklist. (PDF)

S1 Graphical abstract (TIF)

S1 Table Characteristics and main results of included studies on clinical assessments (n = 37) for the determination of leg length discrepancy. (DOCX)

S2 Table Characteristics and main results of included studies on imaging diagnostics (n = 15) for the determination of leg length discrepancy. (DOCX)
