# Supplementary figures and images for: Leg length discrepancy: A systematic review on the validity and reliability of clinical assessments and imaging diagnostics used in clinical practice
Source: PLoS One. 2021 Dec 20;16(12):e0261457. doi: 10.1371/journal.pone.0261457 (PMC8687568; doi:10.1371/journal.pone.0261457)

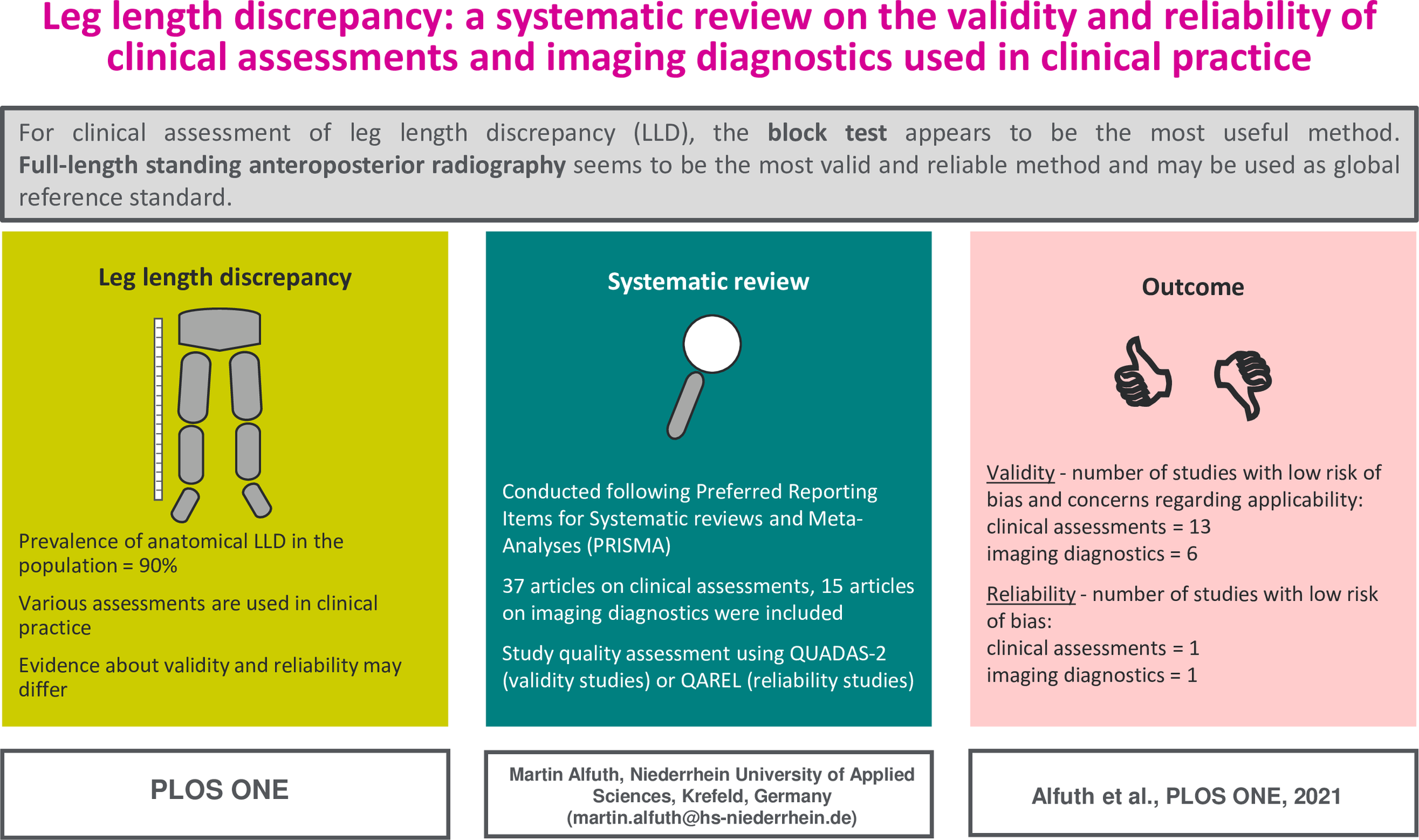

Supplement: S1 Graphical abstract — (TIF) [file pone.0261457.s002.tif]
