# Supplementary material for: Leg length discrepancy: A systematic review on the validity and reliability of clinical assessments and imaging diagnostics used in clinical practice
Source: PLoS One. 2021 Dec 20;16(12):e0261457. doi: 10.1371/journal.pone.0261457 (PMC8687568; doi:10.1371/journal.pone.0261457)
Supplement: S1 Table — (DOCX) [file pone.0261457.s003.docx]

**S1 Table:** Characteristics and main results of included studies on clinical assessments (n = 37) for the determination of leg length discrepancy.

| **Author;**  **Study design** | **Population;**  **Subjects;**  **Examiner** | **Main aim;**  **Test methods;**  **Analysis** | | **Main results** | **Conclusion;  Comment** |  |
| --- | --- | --- | --- | --- | --- | --- |
| Aguilar et al. (2017)  Validity study | Spain  17 subjects [11 females, 6 males with leg length difference (mean age = 22.47, range = 18–27 years)]  Not described | To investigate the relationship between the method measuring the distance between malleolus medialis (DIMG) and lateralis (DEMG) and the ground and other clinical measurements for the clinical diagnosis of leg-length inequality  Distance between malleolus medialis (DIMG) and lateralis (DEMG) and the ground; tape measure method (TMM) [Anterior superior iliac spine (ASIS) – malleolus medialis] and (ASIS – malleolus lateralis); pelvimeter measurement; Palpation Meter (PALM); Foot Posture Index (FPI); navicular drop test  Correlation between the measurement of leg length inequality measured by the distance between malleolus medialis (DIMG) and lateralis (DEMG) and the ground and the other clinical measurements using Pearson correlation coefficient, Spearman’s rank correlation coefficient rho, intraclass correlation coefficient, Cronbach’s α, t – Test, Student´s t – test, Mann-Whitney U test | | All participants completed all tests; the following correlations for the clinical methods using the distance between the malleoli and the ground were found: DEMG correlated substantially with TMM (ASIS – malleolus lateralis) (κ = 0.680), i.e. the larger the distance from ASIS to malleolus lateralis, the larger the DEMG; DIMG was inversely proportional to the magnitude of pronation according to the Foot Posture Index, i.e. DIMG decreased with higher valgus of the foot (κ = -0.605); a significant negative correlation was also demonstrated for the DIMG and the navicular drop test, i.e. values of the navicular drop test increased when DIMG decreased (κ = -0.765); statistically significant differences were demonstrated between the tape measure methods (p = 0.019) | The study revealed that the clinical investigation method of measuring the distance between the malleoli and the ground is the method that correlated the most with the other variables and methods of determing leg length discrepancy; main limitations of the study were that examiner(s) were not described and authors frequently refer to the determination of reliability of methods, although (concurrent) validity of the measurement method was investigated; a reference standard was not involved; further studies are required to confirm the validity and to determine the reliability of the method measuring the distance between the malleoli and the ground for assessing leg length discrepancy |  |
| Aspegren et al. (1987)  Validity study | USA  41 consecutive patients with low back pain (gender and age not adressed)  Doctor (visual assessment); X-ray (radiological technician blinded to the clinical assessment results) | To compare visual assessment of leg length discrepancy by using correction with blocks and standing radiography  Visual assessment of leg length discrepancy using three anatomical landmarks with the patient standing; correction of leg length discrepancy using boards of predefined height (3, 6, 12 and 18 to 25 mm); standing X-ray  Difference between clinical assessment and radiographic examination using analysis of variance (ANOVA, p < 0.05); determination of strength of relationship between methods using Eta (η^2^) | | No significant difference between visual assessment and radiographic measurement was demonstrated (z = 0.128 at p-level of 0.05); a strong relationship between the methods was found (η^2^ = 0.885) | The authors concluded that the visual assessment of leg length discrepancy with correction using boards when indicated, is accurate and comparable to X-ray measurement; however, no coefficients of agreement or correlation between methods were calculated; the description of the patients lacked |  |
| Badii et al. (2014)  Diagnostic accuracy study | Canada  10 males, 10 females; age: 23 – 85 years  One rheumatologist; one physical therapist; one occupational therapist; one scientific assisstant in rheumatology | To evaluate the validity, accuracy and reliability of TMM and block correction method while standing and to compare them to the reference standard radiography of the pelvis  Tape measure method (TMM) (ASIS - malleolus lateralis); radiography of the pelvis;  block correction method (standing lift method)  Interobserver-reliability using intraclass correlation coefficients (ICC) between four examiners; t-test; sensitivity and specificity with a cutoff value of > 5 mm | | All participants were assessed with the TMM, the block correction method, and the radiography of the pelvis; interobserver-reliability for the block correction method was moderate (ICC = 0.737) and for the TMM low (ICC = 0.477); intraobserver-reliability was strong for the block correction method (r = 0.93) and for the TMM (r = 0.75); the mean difference between the block correction method and the radiography was -2 mm (p = 0.051) and between the TMM and the radiography -5 mm (p = 0.007); sensitivity and specificity were 55% and 89% for the block correction method and 45% and 56% for the TMM; wrong identification of the shorter leg occurred in 1/20 subjects for the block correction method and in 7/20 subjects for the TMM | Both clinical methods underestimated the leg-length difference; the block correction method was superior to the TMM in all statistical measures |  |
| Beattie et al. (1990)  Validity study | USA  group one: 10 patients with leg-length difference [mean age = 34.1 (± 11.2) years]  group two: 9 healthy participants [mean age = 26.5 (± 3.7) years]  One experienced examiner who performed all measurements | To evaluate the validity of a tape measure method (TMM) compared to radiography for the full length measurement; the leg-length difference was measured with both methods in both groups  Tape measure method (TMM) (ASIS - malleolus medialis); full leg length radiography using mini-scanograms of the ankle, knee, and hip with the subject in supine position  Validity using intraclass correlation coefficients (ICC) | | The TMM revealed intraclass correlation coefficients of ICC = 0.770 (first measurement) and 0.803 (second measurement) for group one and ICC = 0.359 (first measurement) and ICC = 0.786 (second measurement) for group two, and ICC = 0.683 (first measurement) and ICC = 0.790 (second measurement) for all subjects compared to radiography; mean TMM measurements revealed an ICC = 0.852 for group one, an ICC = 0.637 for group two and an ICC = 0.793 for all subjects | The tape measure method is valid compared to radiography when the mean of two measurements is used and when a significant leg-length difference is present; validity is decreased when measurements are carried out in healthy participants; clinicians should not only rely on TMM measurements for clinical decision making; limitations were that only one examiner performed all measurements, radiography was carried out in supine position, sample size was very small and not representative, individual results of TMM showed deviations up to 2 cm and small leg-length differences (< 5 mm) were often miscalculated |  |
|  |  |  | |  |  |  |
| Betsch et al. (2019)  Retrospective cohort study; validity analysis | Germany  99 patients with hip osteoarthritis following total hip arthroplasty [65 females, 34 males (mean age = 54.4 ± 13.4 years)]; 101 matched healthy volunteers (mean age = 52.6 ± 10.6 years)  Not described | | To investigate and compare the influence of simulated leg length inequality in patients after total hip arthroplasty with a control group of subjects without total hip arthroplasty; correlations between measurement methods were also calculated  Tape measure method (TMM) (ASIS - malleolus medialis); rasterstereography; standard anterior-posterior pelvic radiography (x-rays)  Determination of differences in simulated leg length inequality between groups using t-test (p < 0.05); correlation between measurement methods using Pearson correlation coefficient | Mean simulated leg length inequality (difference between right and left leg) was 0.9 ± 14.8 mm for TMM, 1.2 ± 11.6 mm for X-ray, and 0.9 ± 7.9 mm for rasterstereography; TMM measurement weakly correlated with rasterstereography (r = 0.304) and X-ray measurements of pelvic obliquity (r = 0.285) | All measurement methods demonstrated similar results; however, correlations between methods were weak, indicating a low relationship between direct (TMM) and indirect (rasterstereography; pelvic X-rays) measurement of leg length inequality; as reported by the authors, a limitation was that simulated leg length inequality was analyzed instead of long-standing anatomical leg length inequality | |
| Cleveland et al. (1988)  Validity study | USA  10 subjects [4 females, 6 males (age range = 27 to 55 years)]  Not reported | | Investigation of differences between clinical measurements and radiographic measurements of leg length discrepancy; correlation between the clinical and radiographic measurement methods of leg length discrepancy  Tape measure method (ASIS – malleolus medialis); Standing radiography of the pelvis; postero-anterior radiography in supine position  Differences between clinical measurement and radiographic measurements using t-test (p < 0.05); correlation between measurement methods using linear regression analysis (r^2^) | Using 10 mm as the clinically relevant threshold, radiographic measurement methods of leg length discrepancy revealed perfect correlation (r^2^ = 100) and no significant difference (p > 0.05); clinical measurement and radiographic measurements of leg length discrepancy demonstrated poor to moderate correlations for different thresholds [e.g. 10 mm: r^2^ = 36.64 (standing radiograph); r^2^ = 27.68 (supine radiograph)] and significant differences (p < 0.05) | Based on the results, authors concluded, that radiographic measurements were comparable and superior when compared with TMM for the determination of leg length discrepancy; reliability of measurement methods was not investigated | |
| Cooperstein et al. (2003)  Validity and reliability study | USA  Two healthy males, 1 healthy female (Ages: 37, 28 and 34 years)  One chiropractor | | To determine the concurrent validity of instrumented prone compressive leg checking for the measurement of leg length discrepancy compared to the actual (known) artificially induced leg length inequality using shims; to investigate the intraexaminer-reliability of the prone compressive leg checking  Instrumented compressive leg checking in prone position by measuring changes in foot positions induced by standardized shims, using boot pointers on a metric ruler placed between feet on the examination table  Agreement between measurements of compressive leg checking (observed) and the actual (known) artificially induced leg length inequality using shims by linear regression, Bland-Altman 95% limits of agreement and Lin´s concordance correlation coefficient (CCC); intraexaminer-reliability using the intraclass correlation coefficient (ICC) and Lin´s concordance correlation coefficient (CCC) | One examiner completed 26 possible measurements on all three participants; for all measurements different artificial leg-length inequalities were created (by randomly inserting 0 to 6 1.6 mm shims); for each shim condition there was an excellent test-retest reliability (ICC = 0.85 and CCC = 0.95); the Bland-Altman 95% limits of agreement for the observed versus artificial change in leg-length discrepancy was -5.44 to 5.67; the measured and actual leg length discrepancy shared 87% of variation within measurements and 88% between measurements; the mean measurement error was 1.72 mm for comparisons within measurements and 2.01 mm for comparisons between measurements, respectively | The clinical method of instrumented prone compressive leg checking showed a high accuracy to detect an artificially generated leg-length discrepancy; the clinical relevance has to be investigated by comparing measurements of the compressive leg checking to a gold standard of assessing anatomical leg length discrepancy; the interexaminer-reliability should also be determined, as all measurements were made by one examiner; sample size was very small and generalizability could not be concluded; additionally 11 meaningful limitations were described, challenging the design and results of the study | |
| Cooperstein et al. (2004)  Validity study | USA  29 subjects [12 females, 17 males (mean age = 24 years)]  One experienced chiropractor | | To determine the agreement of instrumented compressive leg checking in prone position (observed) and the actual (known) measure of artificially induced leg length discrepancy using standardized shims  Instrumented compressive leg checking in prone position by measuring changes in foot positions induced by standardized shims, using boot pointers on a metric ruler placed between feet on the examination table  Agreement between measurements of compressive leg checking (observed) and the actual (known) artificially induced leg length inequality using shims by linear regression and Bland-Altman 95% limits of agreement | All participants were examined by one chiropractor; observed and actual measurement of leg length discrepancy shared 86% of the variation; the mean measurement error was 2.7 mm; compressive leg checking (observed leg length discrepancy) was accurate in 86.2% of measurements when compared with actual measurement of leg length discrepancy; conversely, in 13.8 % the leg was described relatively longer between observed and actual leg length discrepancy; the 95 % confidence interval for Bland-Altman limits of agreement was -7.6 to + 5.2 mm; the standard error was 3.2 mm | The compressive leg checking in prone position was considered accurate to determine artificial changes in leg length discrepancy of 2-3 mm; the clinical relevance has to be investigated by comparing measurements of the compressive leg checking to a gold standard of assessing anatomical leg length discrepancy | |
| Cooperstein et al. (2017)  Reliability study | USA  31 healthy students [15 females, 16 males (mean age = 26 years)]    Two chiropractors | | To detect the intraexaminer- and interexaminer-reliability of an instrumented prone compressive leg-length checking; determination of the inter-method reliability compared to the sit-stand test  Instrumented prone compressive leg-length checking; sit-stand test  Intraexaminer- and interexaminer reliability using intraclass correlation coefficient | All subjects were assessed using compressive leg-length check, three times by the first examiner and once by the second examiner; 22/31 subjects completed the sit-stand test; the intraexaminer-reliability was fair to good (ICC = 0.71); 15 subjects were included to determine the interexaminer-reliability; agreement was fair to good (ICC = 0.67); in 18 of 22 subjects (81.8%), who were analyzed for the inter-method reliability, there was an agreement between the two methods regarding the evaluation of the shorter leg or equal leg length; the weighted kappa for inter-method agreement was κ = 0.65 (0.38, 0.91) | Fair to good intraexaminer-reliability and interexaminer-reliability was confirmed for the compressive leg-length checking; a good agreement between compressive leg checking and the sit-stand test was concluded; the leg-length checking method was not compared to a valid reference standard | |
| Cooperstein & Lucente (2017)  Reliability study | USA  43 asymptomatic and symptomatic students [16 females, 27 males (mean age = 25.5 years)]  Two experienced chiropractors with over 30 years of experience in both clinical methods | | To determine the intermethod-reliability between methods of spine and prone leg length inequality assessment  Leg length inequality assessment in prone and supine position  Intermethod-reliability using Cohen’s kappa coefficient | Both chiropractors assessed all subjects for leg length inequality in prone and supine position; the kappa coefficient for intermethod-reliability was κ = 0.16 for all measurements, representing slight agreement;  measurements in a subgroup of 20 subjects where both examiners were confident revealed a kappa value of κ = 0.00; the subgroup where one examiner was confident (18 subjects) demonstrated a kappa value of κ = 0.24; the subgroup where no examiner was confident (5 subjects) revealed a kappa value of κ = 0.55 | The study revealed a poor intermethod-reliability between the leg length inequality assessment in supine and prone position; the authors assumed that positioning the patients in the prone position may have increased, decreased, reversed or offset the observed leg length inequality that was seen in the supine position; these findings challenge both methods and should therefore be rechecked in further studies; furthermore, both methods should be validated using a reference standard before implementation into clinical practice | |
| DeBoer et al. (1983)  Reliability study | USA  40 healthy students (ages = 21 - 35 years)  Three chiropractors | | Investigation of the intraexaminer- and interexaminer-reliability for the clinical measurement of leg-length discrepancy  Measurement of the leg-length in prone position with extended and flexed knees (90°)  Intraexaminer- and interexaminer reliability using intraclass correlation coefficient (ICC) and Pearson correlation coefficient | All subjects were assessed by all three examiners with two measurements for leg-length discrepancy in prone position (extended and flexed leg); the agreement for the method in prone position and extended leg within the three examiners was fair to good (ICCs = 0.52, 0.70 and 0.77); the agreement for the method in prone position and flexed leg within the three examiners was fair (ICCs = 0.64, 0.64 and 0.69); the agreement of the measurement method with the knees extended and flexed between examiners was weak to fair with intraclass correlation coefficients ranging from ICC = 0.23 to ICC = 0.37 and Pearson correlation coefficients ranging from r = 0.24 to r = 0.45 for the measurement with extended knees and from ICC = 0.06 to ICC = 0.34 as well as from r = 0.09 to r = 0.37 for the measurement with flexed knees, respectively | Measurement of the leg-length in prone position with extended and flexed knees (90°) showed fair to good agreement within examiners, however, agreement between examiners was weak to fair; a reference standard was not included in the study; the validity of the methods should be investigated in future studies before using the methods for determining leg length discrepancy in clinical practice  . | |
| Edeen et al. (1995)  Validity study | USA  68 subjects who underwent total hip arthroplasty [39 females; 29 males (mean age = 68.5 years; age range = 34 – 91 years)]  One physician | | To compare clinical measurements of leg length inequality to radiographic measurement; correlations between methods were determined  Tape measure method (TMM) (ASIS – medial malleolus and pubis – medial malleolus); difference in length between the soles of the feet with the subject positioned in supine; block test; standing orthoroentgenographic measurement; anteroposterior views of the pelvis  Determination of differences of the clinical methods compared to orthoroentgenographic measurement using t-test; association between clinical and radiographic measurements using chi-square analysis | Clinical methods correlated with each other 77% to 91% of the time to within 10 mm; clinical measurements correlated with radiographic measurements 55% to 65% of the time to within 10 mm; clinical measurements correlated with orthoroentgenographic measurements 51% to 66% to within 10 mm; radiographic measurements correlated with orthoroentgenographic measurements 62% of the time to within 10 mm | It was concluded that clinical measurements of leg length discrepancy poorly correlated with those carried out with orthoroentgenography and were inaccurate; however, statistical analysis was described superficially, making it difficult to follow; the formulation of the aims of the study varied across the study and was therefore confusing | |
| Farella et al. (2005)  Validity and reliability study | Italy  41 dental students [18 females, 23 males (mean age = 24.5 years; age range = 21 – 34 years)]  Three examiners (one expert chiropractor, one dentist with chiropractic experience, one general dentist) | | To determine the interexaminer-reliability and validity of the leg-length inequality test  Supine leg check by comparing the position of the malleolus medialis and detecting the side of leg-length inequality  Summarization of data agreement using 3 x 3 tables; determination of interexaminer-reliability using proportion agreement (%) and chance-corrected kappa statistics | Leg-length inequalities were identified by all examiners with percentages of 56.1%, 53.6%, and 58.5%, respectively; the proportion of observed agreement between examiners ranged from 0.56 to 0.61; agreement between examiners was low with kappa coefficients ranging from κ = 0.33 – 0.39; Sensitivity for myofascial pain of jaw muscles was 0.43 and for anterior temporomandibular joint disc displacement 0.50; specificity was 0.41 and 0.41, respectively | The authors concluded poor interexaminer-reliability for the leg-length inequality test; validity of the leg-length inequality test as assessment for differentiation between patients with temporomandibular disorder and healthy participants was very low | |
| Friberg et al. (1988)  Validity study | Finland  21 patients with low back pain [2 females, 19 males (mean age = 31 years; age range = 19-80 years)]  Five experienced examiners (three general practitioners and two physiotherapists) | | To evaluate the accuracy and precision of direct and indirect measurements of leg length discrepancy  Direct measurement: tape measure method (TMM) (ASIS – malleolus medialis) with the patient recumbent; indirect measurement 1: estimation of lateral pelvic inclination by palpating the iliac crests for planarity; indirect measurement 2: placement of wooden boards of known thicknesses under the shorter leg until iliac crests were considered level; standing full-weight bearing anteroposterior radiography  Determination of accuracy and precision of leg length discrepancy measurements using calculation of mean error and mean variation; interexaminer-variation using analysis of variance (ANOVA); intraexaminer-variation (not specified) | The observer error for the clinical measurements was high with ± 8.6 mm for direct and ± 7.5 mm for indirect assessment of leg length discrepancy; when the criterion of leg length discrepancy was 5 mm, 53% of measurements were inaccurate; the combined estimate of an intraexaminer-error in measuring leg length discrepancy was 5.5 mm for direct and 5.8 mm for indirect measurement methods | Clinical assessments of leg length discrepancy revealed a wide variation; direct as well as indirect measurement methods frequently disagreed with the “true” leg length discrepancy measured by radiography; correlation coefficients or coefficients of agreement between methods and examiners were not determined | |
| Gross et al. (1998)  Validity and reliability study | USA  22 subjects between the ages of 18 and 55 years [19 females (mean age = 34.8 years), 13 males (mean age = 36,9 years]  Two examiners completed the clinical method; one examiner performed the radiography | | To determine the validity and reliability of assessing functional leg-length inequality using a pelvic leveling device compared to standing radiography  Lift and pelvic leveling device method; standing radiographic measurement  Validity, intratester-reliability and intertester-reliability using intraclass correlation coefficients (ICC) and absolute differences between measurement methods | All participants were measured using the lift and pelvic leveling device method and standing radiography; the value for intratester-reliability of the clinical method was ICC = 0.84 with an absolute mean difference of 0.29 (± 0.52) cm; the value of intertester-reliability of the clinical method was ICC = 0.77 with an absolute mean difference of 0.49 (± 0.46) cm; the value of agreement between the clinical measurement and radiography for examiner one was ICC = 0.64 with an absolute mean difference of 0.58 (± 0.58) cm; the value of agreement between the clinical measurement and radiography for examiner two was ICC = 0.76 with an absolute mean difference of 0.55 (± 0.37) cm; measurement of leg length discrepancy using the clinical method disagreed with radiographic measurements in five subjects for examiner one and in four cases for examiner two | ICC values revealed good intra- and intertester-reliability and moderate validity for the clinical measurements, however, authors concluded an unacceptable reliability and validity; no predefinition of the levels of agreement was provided; the high absolute differences between measurements were considered the main reason for the conclusion | |
| Hanada et al. (2001)  Validity and reliability study | Canada  34 healthy adults (18 males,16 females) at the ages >19 years  two examiners for the clinical method; one radiologist for radiography | | To determine the construct validity, the concurrent validity, and the reliability of the iliac crest palpation and book correction (ICPBC) method  ICPBC method to measure induced leg-length discrepancy;  standing radiography of the pelvis/hip  Construct validity, concurrent validity, intrarater-reliability, and interrater-reliability using intraclass correlation coefficients (ICC); mean difference (± standard deviation) | Intrarater- and interrater-reliability were excellent (ICC = 0.98 and ICC = 0.91, respectively); construct and concurrent validity were moderate (ICC = 0.62 and ICC = 0.76, respectively); the ICPBC method underrated the simulated leg-length discrepancy by a mean difference of 3.8 (± 10.3) mm (p = 0.055) and the radiography by a mean difference of 5.1 (± 8.6) mm (p = 0.043) | The ICPBC method revealed excellent reliability and moderate validity; the ICPBC method is recommended, if there is no history of pelvic deformity and the iliac crest can be simply palpated | |
| Harris et al. (2005)  Validity study | Australia  35 skeletally mature patients with a history of femoral shaft fracture [10 females, 25 males; mean age = 33 (range = 14-76) years]  One surgeon for the measurement using clinical methods; radiologists of the hospital performed computed tomography (CT) measurements | | To investigate the correlation between TMM and block test measurements compared to CT scanogram and patient perception of limp  Tape measure methods (TMM) (ASIS - malleolus medialis);  block test; patient perception of limp; CT scanogram  Correlation between clinical methods and CT using Pearson product moment correlation coefficient (p< 0.05); absolute mean difference | All patients completed the clinical tests; 29/35 performed an additional CT scanogram to evaluate leg length discrepancy and correlation between the methods; significant positive correlations between TMM and block test (p = 0.003), between block test and patient perception of limp (p = 0.042), and between block test and leg length discrepancy (p = 0.004) were found; patient perception of limp correlated positively with the perception of leg length discrepancy (p = 0.047); authors reported, that there was no correlation between CT scanogram and TMM, block test and patient perception of limp as well as leg length discrepancy; the absolute mean difference between clinical measurement and CT was 7.24 (± 7.98) mm with a maximal difference of 40 mm | A positve correlation between clinical measurement methods and patient perception was detected; no correlation between CT scanogram and the other examination methods such as patient perception was reported; the authors concluded that clinical examination methods are more reliable and relevant in practice than a CT scanogram; period of time between clinical examination methods and CT was not described; the senior author performed all clinical measurements; correlation coefficients were not reported, and therefore, strength of correlations were not interpretable; correlation analysis was not comprehensible | |
| Hellsing (1988)  Reliability study | Sweden  999 men (age range = 18-19 years) were included; 613 men completed the second measurement; 547 men completed the third measurement  One examiner (not further described) | | To investigate the reproducibility of leg length inequality screening with the subject in standing position  Assessment of the levels of the anterior and posterior iliac spines by eye judgement during palpation with five defined categories of differences  Strength of correlation between three measurements using contingency correlation (c) and 2 x 2 and 3 x 3 tables; significance of correlations using chi square test | Significant correlations were found between the results of the three examinations with a total agreement of 62-66% (p = 0.0001); correlation coefficient between different types of leg length difference between measurement 1 and 3 was c = 0.529, representing some association between the measurements | A clear conclusion regarding reproducibility of examinations was not provided by the author; a strength of the study was the large sample size, however, a great number of drop-outs have been observed at examination two (n = 386; 38.6%) and three (n = 452; 45.2%) | |
| Holt et al. (2009)  Reliability study | New Zealand  46 volunteers [33 students, 13 patients (ages = 18 - 55 years)]  One chiropractor with seven years of work experience; one chiropractic student | | To evaluate the interexaminer-reliability of a leg length analysis protocol between an experienced chiropractor and a chiropractic student  Leg length analysis in prone position  (straight leg; flexed knees; combined position)  Agreement between the examiners using Cohen‘s kappa coefficient with 95 % confidence interval and percent of agreement | The leg-length analysis in prone position was completed by both examiners for all participants; substantial reliability for the determination of the short leg for the prone position with straight legs (κ = 0.70; 95% CI = 0.49 to 0.92) with 87% agreement was found between both examiners; substantial reliability for the method with flexed knees (κ = 0.65; 95% CI = 0.44 to 0.86) with 83% agreement was observed between the examiners; the combined position revealed less, but also substantial agreement (κ = 0.6; 95% CI = 0.43 to 0.78) with 72% agreement between examiners; in 43% of cases the shorter leg “became shorter” when the knees were flexed to 90 degrees | A good interexaminer-reliability for the leg length analysis in prone position using different positions (knees extended, knees flexed, combined position) was found; further studies are required to evaluate the validity and accuracy of these methods in prone position | |
| Jamaluddin et al. (2011)  Cross-sectional validity and reliability study | Malaysia  48 [35 males, 13 females (mean age = 22.6 ± 15.8 years)]; 22 [15 males, 7 females (mean age = 14 ± 5.46 years)] of the 48 subjects were additionally measured using a CT scanogram  Two experienced surgeons, two experienced radiologists | | To determine the reliability and accuracy of the tape measure method (TMM) compared to CT scanogram  TMM (ASIS – malleolus medialis) with a nearest reading of 5 mm; CT scanogram  Agreement between measurements using intraclass correlation coefficient (ICC) with 95% confidence interval (CI); paired t-test (p < 0.05) for statistical significance of differences | All subjects completed the TMM, 22 of 48 participants completed additional CT scanogram; the TMM- value measured by surgeon I compared to CT scanogram- value measured by radiologist I revealed good agreement (ICC = 0.81); the mean TMM- value of both observers compared to the CT scanogram- value of radiologist I revealed good agreement (ICC = 0.85); interrater-reliability for TMM and for CT scanogram was excellent with ICC = 0.924 and ICC = 0.971, respectively; no significant mean difference was found between the TMM and CT; the mean difference between the TMM- and CT scanogram measurements was 1.95 mm (95% CI: -3.17 mm to 7.07 mm); the mean difference between the TMM measurements of both surgeons was -1.35 mm (95% CI: -3.87 mm to 1.17 mm) | There was a good agreement between TMM and CT scanogram; both methods demonstrated an excellent interrater-reliability; no significant mean difference was found between TMM and CT scanogram; 95% CI was wide | |
| Junk et al. (1992)  Reliability study | Norway  100 adults [56 males (mean age = 33 years, range = 18-66 years); 44 females (mean age = 34 years, range = 16-64 years)]  Two independent examiners | | To determine the reliability of an ultrasound method and the block test and the tape measure method (TMM); comparison of the interobserver-variance between ultrasound method and clinical examination methods  TMM (ASIS – malleolus medialis) with the participant positioned in supine; block test; ultrasound technique  Differences in the distribution of interobserver-variance using t-tests, analysis of variance (ANOVA) and Chi-square test (p<0.05) | All participants completed two measurements using ultrasound and both clinical measurements; mean interobserver-variance for ultrasound was 0.9 (± 2.5; range = - 5-8) mm, for the block test - 1.0 (± 4.8; range = - 15-20) mm and for the TMM - 1.0 (± 5.3; range – 15-10) mm; the interobserver difference >5 mm was significantly higher using the block test compared to ultrasound (p=0.04) and using TMM compared to ultrasound (p<0.001); the difference of leg length discrepancy assessed by ultrasound compared to clinical examination methods was smaller than 10 mm in about 95% of participants | For leg length discrepancy < 5 mm a significant smaller interobserver difference assessed by ultrasound method compared to clinical measurements was observed; two measurements were completed using the ultrasound method and the average value was used for further analysis only for ultrasound measurement; authors aimed to evaluate reliability of the measurement methods, however, no measures of correlation or agreement between examiners and between methods were used; validity was not analyzed; no valid reference standard was used for comparison; examination methods and statistical methods were described insufficiently | |
| Krettek et al. (1996)  Diagnostic accuracy study | Germany  50 subjects (age range = 21 – 85 years)  One examiner | | To evaluate accuracy, correctness and validity of sonographic measurement of leg length discrepancy compared with clinical and radiographic measurements  Tape measure method (TMM (ASIS – malleolus lateralis); block test (5 mm increments); sonographic measurement; teleroentgenography  Accuracy using variance; correctness using difference between clinical and sonographic measurements and radiographic measurement; sensitivity and specificity using contingency table; correlation using regression analysis; responsiveness using defined discrimination intervals | The clinical methods revealed an average variance of -1.2 ± 9.6 mm (tape measure) and -1.0 ± 6.1 (block test) compared to the radiographic measurement; the sonographic measurements demonstrated an average variance of 0.9 ± 2.7 mm compared to the radiographic measurements; sensitivity of clinical measurements to determine a leg length discrepancy of > 5 mm was 58.3% (tape measure) and 62.5% (block test); sensitivity of sonographic measurement to determine a leg length discrepancy > 5 mm was 91.7%; specificity (exclusion of leg length discrepancy ≤ 5 mm) for the tape measure method was 53.9%, for the block test 65.4% and for the sonographic measurement 69.2%; correlation coefficients were r = 0.826 for the tape measure method, r = 0.922 for the block test and r = 0.978 for the sonographic measurement compared to radiographic measurement | The sonographic determination of leg length discrepancy was considered more accurate and reproducible than the clinical methods; therefore, it was favoured for screening, the adjustment of shoes, control of leg lengthening and to render an expert opinion; furthermore, it causes no radiation exposure | |
| Lampe et al. (1996)  Retrospective validity study | Netherlands  190 patients (children); sex and age were not reported  Clinical measurements were performed by one person (not further described); orthoradiography was performed by one of two authors (not further described) | | To compare the assessment of leg length inequality using wooden boards and tape measure method (TMM) and orthoradiography  Wooden boards of different thicknesses placed under the shorter limb until iliac crests were level; TMM (ASIS – medial knee joint line and medial knee joint line - malleolus medialis) with the participant in supine position; orthoradiography  Calculation of mean difference and 95% limits of agreement (LOA) using Bland-Altman analysis; regression analysis | The mean difference between leg length inequality measurements with wooden boards and orthoradiography was 0.09 ± 0.78 cm with 95% LOAs of -1.4 and 1.6; the mean difference between leg length inequality measurements with TMM and orthoradiography was 0.14 ± 0.97 cm with 95% LOAs of -1.8 and 2.1; the predictive value of the site of the main leg length inequality above the knee was 64% and of the site of leg length inequality below the knee 75% for the TMM | It was concluded, that TMM by adding the distances above and below the knee is inadequate for the identification of the main leg length inequality; direct measurements from the ASIS to malleolus medialis may be preferred; although the assessment of leg length inequality using wooden boards was more precise than TMM, an orthoradiography should be completed instead of both clinical methods | |
| Montgomery et al. (1995)  Validity study | USA  33 subjects with low back pain [11 females, 22 males (ages = 25 – 75 years)]  One experienced practitioner | | To investigate the role of oblique-axis sacral torsion in the presence of true or apparent short leg; in the study, the null hypothesis “palpable unilateral sacral border prominence has no relationship to leg length inequality” was tested; the clinical palpation method was compared to a radiography (Egan method)  Palpation of the unilateral sacral prominence to determine leg length discrepancy; radiography of the lower extremity using the Egan method  Accuracy of palpation of the unilateral sacral prominence compared to radiography using 3 x 3 contingency table; correlation between methods using weighted kappa coefficient | 15 subjects were excluded for different reasons; 18 subjects were examined using palpation of unilateral sacral prominence and using radiography; the comparison of results of radiography and palpation of unilateral sacral prominence by using a 3 x 3 contingency table revealed a statistically significant agreement between the methods for determination of leg length discrepancy; all subjects assessed positive for leg length discrepancy using the palpation of an unilateral sacral prominence demonstrated a leg length discrepancy measured by radiography (p < 0.001); an agreement between the side of sacral prominence and the side of the shorter leg was confirmed (p < 0.05); the correlation between both methods was moderate with a weighted kappa coefficient of κ = 0.664 for detecting the presence of a leg length discrepancy and κ = 0.583 for identifying the side of leg length discrepancy | The study revealed a significant correlation between the palpation of unilateral prominence and radiography in terms of an identification of the leg length discrepancy and the side of leg length discrepancy; overall, the study was hard to follow, because a clear design and purpose of the study were not formulated, it was not clear whether a second examiner conducted the radiographic measurements, and statistically significant results were reported inappropriately (p > 0.001 and p > 0.05); further studies with a larger number of subjects are required to confirm the intermethod-correlation and to investigate the interexaminer-reliability and validity of the palpation of the unilateral sacral prominence to determine leg length discrepancy | |
| Neelly et al. (2013)  Validity and reliability study | USA  30 subjects [19 females, 11 males (ages = 18-70 years)]  Two licensed physical therapists with clinical experience | | To assess the validity of the TMM compared to CT scan and the reliability of TMM  Tape measure method (TMM) [anterior superior iliac spine (ASIS) - malleolus medialis];  computed tomography scan (CT)  Validity, intrarater-reliability and interrater-reliability (ICC); minimal detectable difference (95 %) | All subjects completed the TMM and the CT scan; the validity for a single TMM compared to the CT scan showed an ICC of 0.984 for examiner 1 and ICC of 0.978 for examiner 2; the validity from the average of two TMMs was excellent (ICC of 0.992 and 0.990); intrarater- (ICC = 0.990 and 0.985) and interrater-reliability (ICC = 0.991) were excellent; minimal detectable difference (95%) of measurements of each rater was 1.73 cm (rater 1) and 2.11 cm (rater 2) | The TMM is valid compared to CT scan for leg-length discrepancy; excellent interrater- and intrarater-reliability has been demonstrated; leg length discrepancy was not measured, only the leg length | |
| Nguyen et al. (1999)  Reliability study | USA  34 patients between the ages of 28-88 years [23 females, 11 males (mean age = 58.3 years)]  Two chiropractors with 15 years of experience; one supervisor | | To investigate the interexaminer-reliability of the Activator Method with the subject in prone extended position for determination of relative leg-length inequality  Activator Method with the subject in prone extended position  Interexaminer-reliability by organizing data in a 3 x 3 contingency table and calculating total agreement (sum of agreement cells/n x 100%); calculation of the unweighted kappa coefficient | All participants were tested with the Activator Method by two different examiners; results were recorded as left short leg, equal leg length, or right short leg; there was an agreement of 85% between both examiners; the simple, unweighted kappa coefficient was κ = 0.66 | The study revealed a good reproducibility and interexaminer-reliability between both raters for the Acitvator Method; leg lengths were not determined; the validity and accuracy of this method should be examined in further studies | |
| Petrone et al. (2003)  Validity and reliability study | USA  30 subjects (21 females, 9 males) between the ages of 23 and 73 years; asymptomatic group: 15 healthy soldiers (military); 15 patients with confirmed leg length discrepancy  Two physical therapy students for the clinical method; one radiologist for the radiographic measurement | | To determine the validity of the palpation meter (PALM) compared to standing antero-posterior radiograph for leg length discrepancy; to assess the intra- and intertester reliability of the PALM  PALM; radiography  Validity, intra- and intertester reliability using intraclass-correlation coefficient (ICC); calculation of mean and standard error of measurement for PALM-measurements; difference of leg length discrepancy between groups using unpaired t-tests | All 30 participants completed three measurements by two examiners with the PALM and the radiographic measurement by the radiologist; for all subjects the validity estimates of the PALM was excellent to detect height difference of the iliac crest (ICC = 0.90 for examiner 1 and ICC = 0.92 for examiner 2) compared to standing radiography; the PALM was less accurate as an indirect assessment of leg length discrepancy (ICC = 0.76 and ICC = 0.78 for examiner 1 and 2, respectively); intratester-reliability for each examiner was excellent (ICC = 0.97 and ICC = 0.98, respectively); intertester-reliability was considered very good (ICC = 0.88); the mean values for the PALM-, and radiographic measurements did not differ significantly between groups | The PALM method is valid, reliable and accurate to detect iliac crest height in healthy subjects and subjects with leg length discrepancy; as an indirect method for determining leg length discrepancy, the use of PALM showed moderate agreement compared with a pelvic radiography | |
| Piyakunmala et al. (2018)  Validity and reliability study | Thailand  68 patients undergoing total hip arthroplasty subjects [31 females, 37 males (mean age = 51.71 years)]  One examiner (surgeon) for the clinical test; two examiners (orthopaedic surgeons) for the radiographic measurement | | To compare leg length discrepancy measurements using block test (patient’s perception), weight-bearing orthoroentgenography and pelvic radiography by determining difference, correlation, and reliability; sensitivity and specificity were also calculated  Block test; weight-bearing orthoroentgenography; pelvic radiography  Mean difference between measurement methods using t-test (p < 0.05); intraexaminer- and interrater-reliability and correlation of measurement methods using intraclass correlation coefficient (ICC) and concordance correlation coefficient (CCC) | No significant mean differences between the three measurement methods were found; agreement between measurement methods was poor for the comparisons block test vs. orthoroentgenography (ICC = 0.22) and block test vs. pelvic radiography (ICC = 0.28); sensitivity and specificity of block test measurement (patient’s perception) was 60.98% and 48.15% and of pelvic radiography 78.05% and 85.19%, respectively, when using orthoroentgenography as reference standard; the agreement between orthoroentgenography and pelvic radiography was good (ICC = 0.85); the orthoroentgenography and pelvic radiography revealed excellent intra- and interexaminer-reliability (ICC = 0.92 and ICC = 0.90 as well as ICC = 0.88 and ICC = 0.81, respectively); correlation between orthoroentgenography and block test was poor (CCC = 0.21) and between orthoroentgenography and pelvic radiography good (CCC = 0.85) | It was concluded that the block test (patient’s perception) demonstrated poor reliability and correlation as well as low sensitivity and specificity; therefore, orthoroentgenography or pelvic radiography should be preferred for the measurement of leg length discrepancy | |
| Rhodes et al. (1995a)  Validity and reliability study | USA  50 subjects [22 females, 28 males (mean age = 47.9 years)] with low back pain  One experienced chiropractor | | To assess the validity and reliability of leg length discrepancy measurement with the subject in prone and supine position compared to standing radiography  Prone and supine leg check; standing radiography  Determination of predictors of leg length discrepancy in standing radiographic measurement using multiple regression analysis; correlation between prone and supine leg check and standing radiography using Pearson product-moment correlation coefficient; degree of association among subjects using intraclass correlation coefficient (ICC) | The measurement in prone position and the duration of pain were significant predictors of leg length discrepancy in radiographic measurements (p = 0.0002 and p = 0.024, respectively); an almost perfect correlation was found between the first and second measurement in prone position (r = 0.958); correlations between measurements in prone position and radiographic measurement were moderate and ranged from r = 0.572 to r = 0.619; the correlation between the average measurement in supine position and radiographic measurement was poor (r = 0.292); the association for the measurement in prone position was almost perfect (ICC = 0.957) | Validity of leg length discrepancy measurement in supine was poor when compared with radiographic measurement and were not recommended to estimate standing leg length discrepancy in people with low back pain; intraexaminer-reliability of measurement with the subject in prone position was excellent; interexaminer-reliability has to be assessed in future studies | |
| Rhodes et al. (1995b)  Validity study | USA  50 subjects between the ages of 14-80 years [24 females, 26 males (mean age = 44 years)] with low back pain  One experienced chiropractor | | To assess the validity of measuring leg length inequality using prone leg check compared to standing radiography  Prone leg check; standing radiography  Correlation between prone leg check and standing radiography using Pearson product-moment correlation coefficient; difference between methods using t-test for dependent samples | All participants completed a measurement in prone position and standing radiography; the correlation between measurements in prone position and standing radiography was (r = 0.71); in 54% of measurements the difference between clinical method and radiography was within 3 mm; however, in 12 % the wrong leg was observed as the shorter extremity; 76% of measurements were within 6 mm; in 8 % of measurements there was a difference of 12 mm between the methods | Measurement of leg length inequality using prone leg check revealed a strong correlation compared to standing radiography; large differences and some wrong identified shorter extremities limited the validity; furthermore, only one examiner completed all measurements | |
|  |  | |  |  |  | |
| Sayed-Noor et al. (2009)  Validity study | Sweden  139 patients with osteoarthritis [85 females, 54 males (mean age = 67.5 years; age range = 44-89 years)]  Two examiners (one physiotherapist; one radiologist) | | To assess the correlation between the tape measure method (TMM) and radiological methods for determination of pre- and postoperative leg length discrepancy  TMM (ASIS – malleolus medialis) with the patient in supine position; plain radiography of the pelvis and hip joints  Determination of correlations between the TMM and radiographic measurements using Pearson’s correlation coefficient (r) and intraclass correlation coefficient (ICC); mean differences between clinical and radiological measurements using paired sample t-test (p < 0.05) | Weak and fair correlations between TMM and radiological assessment of leg length discrepancy were found preoperatively (r = 0.21, ICC = 0.33) and postoperatively (r = 0.45, ICC = 0.62), respectively; no significant mean difference between postoperative clinical and radiological measurements were observed (p > 0.05) | TMM was not recommended for the assessment of leg length discrepancy in patients scheduled for total hip arthroplasty prior to surgery; furthermore, TMM should also be used with caution post surgery; for planning total hip arthroplasty radiological measurement of leg length discrepancy should be preferred | |
| Schneider et al. (2007)  Reliability study | USA  45 patients with low back pain (ages = 18 - 65 years)  Two chiropractors | | To evaluate the interexaminer-reliability of the prone leg length analysis procedure  Prone leg length analysis with extended or flexed knees or with the head rotated right and left  Level of agreement of the measurements of each condition between the examiners using Cohen’s kappa coefficient with 95 % confidence intervals and percentages of agreement | All participants completed the prone leg length analysis by two examiners; 22 subjects additionally completed a head rotation; reliability between the examiners to detect the shorter leg was good (κ = 0.65) with an agreement of 82%; the reliability of determining the amount of leg length difference was weak (κ = 0.22) with an agreement of 62% agreement; the side of patients’ reported pain was not significantly correlated with the side of the short leg measured by each examiner; measurements using head rotation right and left revealed poor reliability (50% and 45.5%); when the knees were flexed, there was 93% agreement between examiners that the short leg “became longer” | Good interexaminer-reliability for the prone leg length analysis procedure to detect the shorter leg was concluded, however, reliability was poor for quantifying the accurate amount of leg length difference; determining the change of leg length by head rotations was poorly related between examiners; both examiners strongly agreed in determining the “elongation” of the short leg when the knees were flexed; the prone leg length analysis procedure should be validated using a reference standard in further studies | |
| Schwartzbauer & Hart (2011)  Reliability study | USA  30 healthy student volunteers [13 females, 17 males (ages not described)]  Three experienced chiropractors | | To investigate the interexaminer-reliability of five different clinical methods for assessing leg length inequality  Leg length examination in 1) prone position, 2) prone position with cervical rotation, 3) prone position and 90° flexion of the knees, 4) prone position with hip extension, and 5) supine position  Agreement between measurements of examiners using Cohen’s kappa coefficient and percent values | Low and not significant agreements between measurements of the examiners were demonstrated for the positions 1, 2, 3, and 5 (range of κ- values = -0.051 to 0.184); thereby, percent agreements ranged from 26.6% (position 1 to 46.6% (position 2); measurements with the subject in prone position and hip extension (position 4) revealed a significant moderate agreement between the examiners with a kappa value of κ = 0.482 (p < 0.001) and a percent agreement of 73.3% | Only the leg length examination with the subject in prone position with hip extension revealed a moderate interexaminer-reliability; no reference standard was included in the study; no training session for the examiners that could have increased consistency of measurements were performed before the study conduction; further limitations that could have influenced results were listed by the authors, such as different examination tables, lack of randomization of the order of examiners etc.; the clinical methods used have not been described in detail, hindering replication of measurements; the validity, accuracy and reliability of each position of the method used in this study should be investigated in future studies | |
| Shambaugh et al. (1988)  Reliability study | USA  26 chiropractic students (gender distribution and ages not described)  Five examiners | | To examine the intraobserver- and interobserver-reliability of the Derifield-Thompson test for the determination of leg length inequality in prone position  Derifield – Thompson test for leg length inequality in prone position; Derifield – Thompson test for leg length inequality in prone position with cervical adjustment using Pierce-Stillwagon technique; Derifield – Thompson test for leg length inequality in prone position with gluteal massage  Analysis of variance (ANOVA); t-tests | All subjects were assessed using the Derifield-Thompson test by five examiners; subsequently the subjects were assigned to three groups: no treatment (group 1), cervical adjustment using the Pierce-Stillwagon technique (group 2), gluteal massage (group 3); after completing the treatment, all subjects were tested with the Derifield-Thompson test by the five examiners again; all examiners found significant changes in leg length inequality by additional cervical rotation to the left or right side (p = 0.001); the treatments (adjustment using Pierce-Stillwagon or gluteal massage) had no significant effect on leg length inequality | Authors concluded that leg length inequality could reliably be measured using the Derifield-Thompson test; it was reported that a change of leg length inequality could be identified by head rotation; the treatments (adjustment using Pierce-Stillwagon or gluteal massage) did not lead to a significant change in leg length inequality; no reference standard was used; there was a small sample size within groups; correlations between measurements of examiners were not calculated; the validity, accuracy and reliability should be analyzed compared to a reference standard in further studies | |
| Terry et al. (2005)  Reliability study | USA  16 children/adolescents with anatomical leg length difference [mean age = 9.98 (range = 1.5 - 19) years]  One pediatric orthopaedic surgeon, one pediatric orthopaedic fellow, one orthopaedic resident, one physical therapist | | To assess the interobserver- and intraobserver-variability of the tape measure method (TMM), block measurements and radiography for the determination of lower limb length discrepancy  TMM (ASIS - malleolus medialis);  TMM (ASIS - malleolus lateralis);  block measurement; slit scanogram  Intraobserver- and interobserver-variance (reliability) using  intraclass correlation coefficients (ICC) | TMM (ASIS - malleolus medialis), (ASIS - malleolus lateralis) and block measurement revealed an excellent intraobserver-variance (ICC = 0.78, ICC = 0.88, ICC = 0.86) and interobserver-variance (ICC = 0.8, ICC = 0.83, ICC = 0.83); the absolute mean difference for the intraobserver-variance was 1.07 cm (ASIS – malleolus medialis), 0.89 cm (ASIS – malleolus lateralis), 0.81 cm (blocks); the absolute mean difference for the interobserver-variance was 1.03 cm (ASIS – malleolus medialis), 1.11 cm (ASIS – malleolus lateralis), and 1.01 cm (blocks); intraobserver-variance of the indirect slit scanogram was ICC = 0.94 with a mean difference of 0.58 cm and of the direct slit scanogram ICC = 0.99 with a mean difference of 0.13 cm; interobserver-variance of the indirect slit scanogram was ICC = 0.89 with a mean difference of 0.7 cm and of the direct slit scanogram ICC = 0.98 with a mean difference of 0.28 cm | All three clinical measurements demonstrated excellent reliability, however, absolute mean differences were high; the direct slit scanogram was the most reliable measurement with the lowest mean differences | |
| Woerman & Binder-MacLeod (1984)  Diagnostic accuracy study | USA  Three males, two females (age: not specified)  Twenty physical therapists of varying experience | | To investigate the accuracy and precision of five clinical methods for the evaluation of leg-length discrepancy compared to radiography; in three phases and two measurement sessions all five test methods were performed by all physical therapists in each patient  Tape measure methods (TMM), i.e. direct measurements (ASIS - malleolus medialis, ASIS - malleolus lateralis, umbilicus - malleolus medialis, xiphosternum - malleolus medialis); block correction method, i.e. indirect measurement; split scanography (hips, knees, and ankles); radiography of the pelvis  Accuracy and precision of TMMs compared to radiography using absolute mean differences (± standard deviation); significance of accuracy and precision among methods using t- and F- statistics (α = 0.05) | The block correction method revealed the least mean difference [-0.2196 (± 2.6) cm]; TMM (ASIS - malleolus medialis) revealed mean differences ranging from 0.306 (± 1.079) cm to 1.165 (± 0.942) cm; TMM (ASIS - malleolus lateralis) revealed mean differences ranging from 0.294 (± 2.483) cm to 0.898 (± 0.717) cm; TMM (umbilicus - malleolus medialis) and TMM (xiphosternum - malleolus medialis) demonstrated the highest mean differences [0.418 (± 0.988) cm and 1.987 (± 1.62) cm, respectively] | The block correction method was the most accurate and precise method of all clinical tests; from the direct methods, the tape measurement method with the anatomical landmarks ASIS - lateral malleolus was the most accurate and precise method; tape measure methods showed high mean differences, indicating that their use should be considered with caution | |
| Woodfield et al. (2011)  Reliability study | USA  50 subjects [29 males, 21 females (mean age = 28.8, range = 12 - 67 years)]  Two chiropractors | | To quantify interexaminer-reliability of a standardized supine leg check procedure for the screening of leg length inequality  Supine leg check procedure for determining leg length inequality  Agreement between measurements of both examiners using quadratic weighted Kappa statistic with 95 % confidence interval, Gwet`s first-order agreement coefficient and Bland Altman plot | All participants were tested for leg length inequality with a supine leg length check procedure and tactile and visual control by both chiropractors; the agreement between the examiners was moderate (κ = 0.44; 95% CI = 0.21 - 0.67); the mean absolute difference was 0.43 cm (0.169 inch); 32% of ratings of examiners were in perfect agreement, 58% within around 3.2 mm, 72% within around 4.8 mm, 92% within 9.5 mm and 100% within 12.7 mm; the Bland Altman plot showed possible heterogeneity in reliability; in 80% of subjects, the examiners identified a leg length difference of at least around 3.2 mm (first order coefficient = 0.76) | The results revealed a moderate reliability for the measurement of leg length differences; a good reliability for the detection of a leg length inequality was concluded; further studies are required to investigate the interexaminer- and intraexaminer-reliability; the accuracy of the clinical method and the validity compared to imaging diagnostics was not assessed | |
