# Supplementary material for: Leg length discrepancy: A systematic review on the validity and reliability of clinical assessments and imaging diagnostics used in clinical practice
Source: PLoS One. 2021 Dec 20;16(12):e0261457. doi: 10.1371/journal.pone.0261457 (PMC8687568; doi:10.1371/journal.pone.0261457)
Supplement: S2 Table — (DOCX) [file pone.0261457.s004.docx]

**S2 Table:** Characteristics and main results of included studies on imaging diagnostics (n = 15) for the determination of leg length discrepancy.

| **Author;**  **Study design** | **Population;**  **Subjects;**  **Examiner** | **Main aim;**  **Test methods;**  **Analysis** | **Main results** | **Conclusion;  Comment** |
| --- | --- | --- | --- | --- |
| Clavé et al. (2018)  Reliability study | France  112 patients with a history of major hip surgery, hip osteoarthritis, or developmental dysplasia (age: not described)  Two independent examiners (not further described) | To investigate and compare the reliability of the automatic and manual 3D mode of the EOS^TM^-system  3-dimensional (3D) radiography using the EOS^TM^-system with the subject standing in a ‘not conventional’ position  Intra- and interexaminer-agreement of preoperative measurements with the EOS^TM^- system using intraclass correlation coefficient (ICC) with 95% confidence interval (CI); significance of differences of measurements using the automatic 3D mode versus the manual 3D mode using t-test | For total leg length measurement, the difference between manual 3D mode and automatic 3D mode was 0.5 mm and not significantly different; the interexaminer-reliability for total leg length measurement using the manual 3D mode was almost perfect [ICC = 0.997 (95% CI: 0.995-0.998)]; the intraexaminer-reliability for total leg length measurement using the manual 3D mode was almost perfect as well [ICC = 0.998 (95% CI: 0.996-0.999)]; the interexaminer-reliability for total leg length measurement using the automatic 3D mode was not calculated, however, it was almost perfect for femoral, tibial, and lower-limb lengths (range: ICC = 0.986 to ICC = 0.998); the intraexaminer-reliability for total leg length measurement using the automatic 3D mode was not calculated, however, it was almost perfect for femoral, tibial, and lower-limb lengths as well (range: ICC = 0.995 to ICC = 0.998) | It was concluded, that leg length measurements with 3D radiography using the EOS^TM^-system was highly reproducible; the manual as well as the automatic 3D mode method revealed similar results of leg length measurements without significant differences; validity of the EOS^TM^-system was not evaluated in this study |
| Guggenberger et al. (2014)  Reliability study | Swiss  51 patients with severe knee osteoarthritis scheduled for total knee replacement [29 females, 22 males (mean age = 68.8 years; range = 43-92 years)]  Two experienced musculoskeletal radiologists | To compare the measurements of limb length using biplanar linear radiography, CT-scanogram in supine position, and upright full-length radiography  Biplanar linear radiography; computerized tomography scanogram (CT-scanogram) in supine position; upright full-length radiography  Mean difference of limb length measurement and interreader-agreement using Bland-Altman analyses with 95% confidence interval and t-test | No significant mean difference was found for the comparison of limb length measurement between CT in supine and full-length radiograph; there was a significant mean difference of 2.7 ± 5.5 mm between limb length measurement using CT in supine compared to upright biplanar linear radiography (p < 0.05); there was a significant mean difference of 5.4 ± 13 mm between limb length measurement using upright biplanar linear radiography compared to biplanar linear radiography (p < 0.05); Bland-Altman analyses revealed high interreader agreement among all methods with no significant differences between readers; the highest agreement was observed for the biplanar linear radiography with a mean difference of -0.6 ± 2.8 mm for the standard and -0.5 ± 3.4 mm for the composed limb length measurement | All measurement methods revealed similar results and can be used alternatively for the measurement of limb length; biplanar linear radiography was superior to the other methods regarding interreader agreement; coefficients of agreement or correlation between both methods were not calculated |
| Jensen et al. (2017)  Retrospective comparative validity and reliability study | Denmark  22 [14 girls, 8 boys (mean age = 12.8 years; age range = 10 - 15 years)]  Two experienced radiologists | To investigate, whether preview images differ from diagnostic images using the EOS^TM^- imaging system for the measurement of leg length  Bi-planar imaging system (EOS^TM^) with the subject standing  Comparison between leg length measurements via preview images and diagnostic images using t-test; consistency of measurements between and within examiners using intraclass correlation coefficient (ICC) and Bland-Altman limits of agreement (LOA) | There was no significant difference between preview images and diagnostic images for the determination of leg length (rater 1 = 0.01 cm, rater 2 = 0.03 cm, p > 0.05); excellent inter- and intrarater-reliability was observed for preview (interrater-reliability for the total leg: ICC = 0.997, LOA = -0.89 to 1.10; intrarater-reliability examiner 1 and 2 for the total leg: ICC = 0.997 and 0.998, LOA = -1.05 to 1.03 and -0.94 to 0.66, respectively) and diagnostic images (interrater-reliability for the total leg: ICC = 0.998, LOA = -0.62 to 0.79; intrarater-reliability examiner 1 and 2 for the total leg: ICC = 0.999 and 0.999, LOA = -0.13 to 0.47 and -0.52 to 0.36, respectively) | Measurement of leg length via the preview images as well as the diagnostic images of the bi-planar imaging system EOS^TM^ was considered reliable with no differences between each other; however, it was critically noted that this weight-bearing method could slightly differ from measurements using the gold standard computerized tomography scanogram (CT) with the subject positioned in supine; agreement between preview and diagnostic images was not determined |
| Khakharia et al. (2011)  Retrospective reliability and validity study | USA  40 patients with either leg length discrepancy or lower leg deformity (age range = 30 to 60 years)  Two examiners (one medical doctor, one physician assistant) | To determine the intrarater- and interrater reliability of measuring leg length discrepancy or leg deformity using soft-copy images of the soft-copy images of the picture archiving and communication system (PACS) and using hard-copy images from radiography; agreement between measurement methods was also evaluated  PACS; hard-copy images from radiography  Agreement and consistency of measurements using intraclass correlation coefficients (ICC) with 95% confidence interval | Intrarater- reliability of measurements of leg length discrepancy using soft-copy images of PACS and using hard-copy radiographs was excellent (range: ICC = 0.996 to ICC = 0.997 and ICC = 0.973 to ICC = 0.993, respectively); interrater-reliability of measurements of leg length discrepancy using soft-copy images of PACS and using hard-copy radiographs was excellent (ICC = 0.984 and ICC = 0.963, respectively); the agreement between measurements using soft-copy images of PACS and hard-copy radiographs was excellent for both raters (ICC = 0.987 and ICC = 0.993, respectively); overall, confidence intervals were narrow | Measurements of leg length discrepancy using soft-copy images of PACS and hard-copy radiographs are similarly reliable and comparable; therefore, PACS is a confidential method and can also be used in clinical practice |
| Kjellberg et al. (2012)  Validity and reliability study | Sweden  10 consecutive patients with primary hip osteoarthritis who underwent total hip arthroplasty [mean age = 76 (65 to 88) years]  Four examiners (one senior orthopaedic resident, one specialist orthopaedic surgeon, one senior radiology resident, one specialist radiologist) | To assess the interobserver- and intraobserver-reliability of length discrepancy measurements using radiographs of the pelvis; to determine the agreement of measurements using radiographs of the pelvis compared to CT-scanogram  Postoperative leg length discrepancy measurements using anteroposterior radiographs of the pelvis; computerized tomography scanogram (CT-scanogram)  Interobserver- and intraobserver-reliability of measurements via radiographs of the pelvis using  intraclass correlation coefficients (ICC) with 95% confidence interval and Pearson correlation coefficient; agreement between measurements via radiographs of the pelvis and CT-scanogram using  intraclass correlation coefficients (ICC) with 95% confidence interval and Pearson correlation coefficient | The agreement between measurements using radiographs and CT-scanogram was moderate to excellent for the examiners (range: ICC = 0.58 to ICC = 0.82; r = 0.44 to r = 0.71) with large 95% confidence intervals; ICC coefficients ranged from ICC = 0.79 to ICC = 1.00, representing excellent interobserver-reliability between all examiners; ICC coefficients and Pearson correlation coefficients were ICC = 0.88 and r = 0.88 as well as ICC = 0.90 and r = 0.90 within examiner 4 and examiner 1, respectively, respresenting excellent intraobserver-reliability | The authors concluded, that reliability of postoperative leg length discrepancy measurements using anteroposterior radiographs of the pelvis was excellent, however, agreement with CT-scanogram was only moderate; therefore, it should be used cautiously in clinical practice and CT-scanogram should be preferred when further interventions, e.g. an implant revision, are required |
| Konermann & Gruber (2002)  Reliability study | Germany  50 healthy subjects [19 females, 31 males (mean age = 29.8 years)]; 6 corpses  Two examiners (not further described) | To investigate the reproducibility and accuracy of leg length measurement using ultrasound  Ultrasound (measuring points on the hip, knee, and upper ankle)  Intra- and interobserver reliability using t-test | The mean differences between the first and second measurements of total leg length, length of the femur and length of the tibia ranged between 0.01 cm and 0.06 cm for examiner 1 and between 0.02 and 0.11 cm for examiner 2; the mean difference of total leg length measurement at the corpses was 0.08 cm | A good reproducibility of sonographic determination of total leg length, length of the femur and length of the tibia was concluded; coefficients of correlation or analyses of agreement between examiners were not calculated; a reference standard was not included; overall, less data of measurements were presented |
| Lazennec et al. (2016)  Nonrandomized, prospective study; reliability analysis | France  70 patients with hip or knee pain due to degenerative joint disease (age range = 18 to 80 years)  Senior author/surgeon (medical doctor); second examiner not mentioned | To investigate if 2-dimensional and 3-dimensional radiographic measurements differ significantly; interexaminer-agreement of the methods was also calculated  2- and 3-dimensional radiographic imaging using the bi-planar imaging system (EOS^TM^) with the subject standing  Comparison of interexaminer-agreement between 2 dimensional- and 3 dimensional methods using intraclass correlation coefficient (ICC) with 95% confidence interval and Bland-Altman analysis; significance of differences between 2-dimensional and 3-dimensional measurements of functional and anatomical leg lengths et al. using t-test | The difference between 2-dimensional and 3-dimensional functional length measurement was 2 mm and anatomical length measurement 6 mm; agreement between examiners was almost perfect for all measurements using 2-dimensional and 3-dimensional imaging (range: ICC = 0.91 to ICC = 0.99 and ICC = 0.92 to ICC = 0.99, respectively); thereby, ICC values for functional and anatomical leg lengths were ICC = 0.99 for both methods; Bland-Altman analysis revealed an interexaminer-agreement of 0.0 ± 3.5 for 2-dimensional functional and 0.2 ± 3.0 for 2-dimensional anatomical measurements as well as an interexaminer-agreement of -0.3 ± 2.9 for 3-dimensional functional and -0.2 ± 3.7 for 3-dimensional anatomical measurements | Both, 2-dimensional and 3-dimensional measurements of functional and anatomical leg lengths were considered accurate; interexaminer-agreement was almost perfect for both methods; it was noted that the senior surgeon performed all measurements; however, interexaminer-agreement was assessed and the second examiner was not mentioned; agreement between 2-dimensional and 3-dimensional measurements was not calculated |
| Meermans et al. (2011)  Reliability study | United Kingdom  52 patients with osteoarthritis of the hip undergoing total hip arthroplasty [23 women, 29 men (mean age = 68.4 years; range = 49 - 78 years)]  Two examiners (medical doctors) | To investigate the difference between measurements of leg length discrepancy using pelvic radiography and full-leg radiography (true leg length discrepancy); to evaluate and compare the intraexaminer- and interexaminer-reliability of radiographic measurements of the pelvis  Pelvic radiography; standing anterior-posterior full-leg radiography  Significance of the mean difference between preoperative measurements of leg length discrepancy using pelvic radiography and full-leg radiography using analysis of variance (ANOVA); intra- and interexaminer-agreement of radiographic measurements of the pelvis using kappa coefficient (κ) | A statistically significant mean difference of the measurement of true leg length discrepancy using full-leg radiography and the measurement using pelvic radiography was found for the biischial-line to the tip of the lesser trochanter (- 2.6 mm) and for the biischial-line to the center of the femoral head (- 2.3 mm) (p < 0.01); intraexaminer-agreement was almost perfect (κ = 0.84 to κ = 0.93) and interexaminer-agreement was substantial to almost perfect (κ = 0.66 to κ = 0.87) for all methods of pelvic radiography | It was concluded, that the method using the pelvic reference interteardrop line and center of the femoral head was the most accurate method for the preoperative assessment compared to the measurement of true leg length discrepancy using full-leg radiography; reliability was not determined for the measurement using full-leg radiography; furthermore, correlation between methods of pelvic radiography and full-leg radiography was not computed |
| Poutawera & Stott (2010)  Reliability study | New Zealand  26 children with various orthopaedic diagnoses (median age = 11 years; age range = almost 2 – almost 18 years)  Seven physicians (five orthopaedic surgeons, one orthopaedic fellow, one consultant radiologist) | To determine intra- and interexaminer test-retest- reliability of CT scanograms  Anteroposterior computed tomography (CT) scanograms with the subjects positioned supine    Calculation of intra- and interexaminer test-retest reliability using intraclass correlation coefficient and Bland-Altman analysis with 95% limits of agreement (LOI) | Almost perfect test-retest reliability of total limb length measurements was found for all examiners (ICC > 0.995; range of mean differences between measurement 1 and 2 = -1.2 mm to 1.2 mm with a LOI- range from -9.9 mm to 8.7 mm); interexaminer- reliability of total limb length measurement was almost perfect as well (ICC = 0.997); Bland-Altman analysis revealed LOIs of ± 0.7 cm for the total limb length measurement | Although agreement of repeated measurements was almost perfect, authors concluded, that outlying measurement errors > 1 cm in the study indicate, that CT scanograms should be carried out more than once and should be double-checked by the surgeon before surgery in children; a reference standard was not included |
| Rannisto et al. (2011)  Reliability and validity study | Finland  Twenty healthy students [18 females, 2 males (mean age = 23 years; range = 19 to 35 years)]  Two physiotherapists; one radiologist | To investigate the intratester- and intertester-reliability of the laser-based ultrasound method and its agreement with the radiography  Laser-based ultrasound; standing radiography  Intratester- and intertester-reliability of ultrasound and radiography as well as agreement between ultrasound and radiography using intraclass correlation coefficients with 95% confidence interval (CI), Bland Altman plots with 95% limits of agreement (LOA), mean of differences and standard deviation of the difference | Intratester-reliability of the ultrasound method was almost perfect for both testers with ICC- values ranging from ICC = 0.994 (95% CI: 0.985 - 0.998) to ICC = 0.996 (95% CI: 0.990 - 0.998), mean differences of 0.40 mm and 0.56 mm, standard deviations of the differences of 3.14 mm and 2.61 mm and 95% LOAs ranging from -5.88 mm to 6.68 mm and -4.66 mm to 5.78 mm; intertester-reliability was almost perfect as well with an ICC = 0.994 (95% CI: 0.988 – 0.997), a mean difference of 0.35 mm, a standard deviation of the differences of 3.01 mm and a 95% LOA ranging from -5.67 mm to 6.37 mm; agreement between ultrasound and radiography was almost perfect with an ICC = 0.971 (95% CI: 0.93 – 0.99), a mean difference of 0.8 mm, a standard deviation of the differences of 2.2 mm, and a 95% LOA ranging from -3.66 to -5.30 mm | It was concluded, that reliability and agreement between the laser-based ultrasound method and radiography were almost perfect; the ultrasound method is non-invasive and could be used alternatively to radiography; furthermore, it was considered quick and easy to perform, either for nurses or physiotherapists; however, a small sample of convenience was included in this study, challenging the generalizability of results; moreover, only healthy subjects were measured |
| Reina-Bueno et al. (2017)  Reliability and validity study | Spain  47 consecutive subjects [23 females, 24 males (mean age = 31.47 years; age range = 17-61 years)]  Two examiners (author; the second examiner was not further described) | To compare the upright full-length radiographic measurement for load-bearing lower limbs with anteroposterior load-bearing hip radiography; reliability and validity were also determined  Upright full-length radiographic measurement for load-bearing lower limbs; anteroposterior load-bearing hip radiography  Intra- and interobserver reliability of measurements using intraclass correlation coefficient (ICC); correlations between measurements using Pearson correlation coefficient; Comparison of means of different measurements using t-test (p < 0.05) | Overall, an excellent to almost perfect intraexaminer- and interexaminer-agreement was demonstrated for all measurements with ICC- values ranging from ICC = 0.952 to ICC = 0.999 (intraexaminer-agreement using difference in height between the central point of the head of the left and right femurs as reference point) and ranging from ICC = 0.789 to ICC = 0.998 (interexaminer-agreement using the highest part of the head of the femur at the medial point of the articular face of the homolateral tibia or using the difference in height between the highest point of the head of the left and right femurs as reference points); correlations between both measurement methods were strong ranging from r = 0.703 to r = 0.988 (correlation between methods using the difference in height between the central point of the head of the left and right femurs or the difference in height between the highest point of the head of the left and right femurs as reference points) | It was summarized that the anteroposterior load-bearing hip radiography was a valid and reliable method to determine leg length discrepancy; both methods were recommended for clinical use of leg length discrepancy assessment |
| Riad et al. (2010)  Cross-sectional reliability study | Sweden  44 patients with spastic hemiplegic cerebral palsy [20 females, 24 males; median age = 17.6 (range = 13 - 23) years]; 14 healthy controls [median age = 17.4 (range = 13.1 – 22.0) years]  Two experienced medical doctors | To explore the amount of leg length discrepancy in patients with spastic hemiplegic cerebral palsy; intraexaminer- and interexaminer-reliability was additionally assessed  Magnetic resonance imaging (MRI)  Differences in leg length within the group of patients using t-test; intraexaminer- and interexaminer reliability using intraclass correlation coefficient with 95% confidence interval (CI) | There was a significant difference of the length of the calcaneus, talus and the tibia between the hemiplegic and the non-affected side (p < 0.01); the five measured bones (calcaneus, talus, tibia, femur, and pelvis) did not significantly differ between the right and left side in the control group; here, the mean total leg length discrepancy was 0.2 mm with a range of 0 to 9.9 mm); intraexaminer-reliability and interexaminer-reliability were excellent to almost perfect for all measured bones (ICC = 0.84 to 0.99 for both) | The measurement of leg length discrepancy including five bones of the leg using MRI revealed excellent reliability in young patients with spastic hemiplegic cerebral palsy; the sample size of the control group was considerably smaller than that of the experimental group; furthermore, little data of the control group were reported |
| Sabharwal et al. (2006)  Retrospective validity and reliability study | USA  111 subjects [65 males, 46 females (mean age = 18.8 years; age range = 9 months to 73 years)]  One examiner (medical doctor) | To compare the results of leg length discrepancy measurements using scanogram and standing anteroposterior radiography  Scanogram using computed radiography with the subject in supine position; standing anteroposterior radiography  Intrarater-reliability using intraclass correlation coefficient (ICC); difference between measurements of leg length via standing anteroposterior radiography and scanogram using analysis of variance and t-tests; agreement between the measurements of leg length discrepancy via anteroposterior radiography and scanogram using correlation coefficient (r) and Bland-Altman 95% limits of agreement (LOA); further analyses included Fisher’s exact test and Cochran-Armitage trend test as well as simple regression analysis to account for various variables potentially influencing measurements | Excellent intrarater-reliability was previously calculated for both methods using seventy sets of each imaging technique (scanogram: ICC = 0.995; standing anteroposterior radiography = 0.980); the measurements of 94 subjects could reliably analyzed for the retrospective analysis of data; a significant correlation of leg length discrepancy determination of the total leg between both methods was found (r = 0.96, p < 0.01); the mean difference of leg length discrepancy measurements between the methods was 0.5 ± 0.5 cm; the mean Bland-Altman 95% limits of agreement were 0.5 to 1.5 cm; the difference of leg length discrepancy assessments between the methods was ≤ 0.5 cm in 64% of subjects and ≤ 1 cm in 85% of subjects | A good agreement between both methods was concluded; a scanogram may underrate the magnitude of leg shortening, because it does not include the determination of foot height; with the use of known lifts that are placed beneath the shorter leg to level the pelvis, leg length discrepancy can be determined more accurately by the use of a standing full-length radiography; therefore, the standing anteroposterior radiography should be the first choice for the initial determination and follow-up of patients with leg length discrepancy; only one examiner performed all measurements and determination of interrater-reliability of the methods should be carried out in future studies |
| Sabharwal et al. (2007)  Retrospective reliability study | USA  70 subjects [39 males, 31 females (mean age = 20 years; age range = 9 months to 73 years)]  Five observers (two fellowship-trained pediatric orthopaedists, two musculoskeletal radiologists, one senior orthopaedic resident) | To evaluate the intrarater- and interrater-reliability of the scanogram using computed radiography with the subject in supine position and standing anteroposterior radiography for the assessment of leg length discrepancy  Scanogram using computed radiography with the subject in supine position; standing anteroposterior radiography  Intra- and interrater-reliability using intraclass correlation coefficient (ICC); mean absolute difference with 95% confidence interval | The mean extent of leg length discrepancy was 23 mm (range = 1 to 96 mm) measured by the scanogram and 21 mm (range = 0 to 98 mm) measured by standing anteroposterior radiography; the intraclass correlation coefficients for the intrarater-reliability ranged from ICC = 0.975 to ICC = 0.995 with a mean absolute difference from 1.5 to 2.6 mm for the measurements of all raters using the scanogram and from ICC = 0.939 to 0.996 with a mean absolute difference from 1.5 to 4.6 mm for the measurements using standing anteroposterior radiography, indicating excellent intrarater-reliability; interrater-reliability was also excellent with intraclass correlation coefficients and mean absolute differences of ICC = 0.979 and 2.6 mm for the scanogram and ICC = 0.968 and 3.0 mm for the standing anteroposterior radiography | Intra- and interrater-reliability was excellent for both methods and results from both methods were comparable; authors concluded that standing anteroposterior radiography should be preferred because it allows for a broader assessment of the legs in patients with leg length discrepancy who may additionally have angular deformities |
| Tipton et al. (2016)  Validity and reliability study | USA  95 subjects [60 females, 35 males (mean age = 68.0 ± 10.5; range = 34-90 years)]  Two examiners (not further described) | To determine the validity of the pelvic measurement technique using radiographic views of the pelvis and proximal femur compared with full-length standing anteroposterior radiograph for the measurement of preoperative leg length discrepancy  Full leg length anteroposterior radiography using different measurement techniques for the determination of leg length discrepany  Intra- and interrater-reliability of different measurement techniques to determine leg length discrepancy using intraclass correlation coefficient (ICC); comparison between different measurement techniques using radiographic views of the pelvis and proximal femur and full-length standing anteroposterior radiograph using t-test | There were significant differences between the average leg length discrepancy determined by radiographic views of the pelvis and full-length standing antero-posterior radiographs of 5.86, 4.75, 4.55, and 3.73 mm (p < 0.001); moderate to almost perfect intrarater-reliability (ICC = 0.62 to 0.95) was demonstrated for all measurement techniques, with the full-length standing anteroposterior radiography being the most reliable technique (ICC = 0.95); Fair to strong interrater-reliability (ICC = 0.29 to 0.78) was observed for all measurement techniques, with the full-length standing anteroposterior radiography and the view from the bi-ischial line to the center of the femoral head being the most reliable techniques (ICC = 0.78) | The results from the study revealed that measurements of leg length discrepancy using radiographic views of the pelvis and the proximal femur significantly differ from those measured by full-length standing anteroposterior radiography; interrater-reliability for the radiographic views was fair to strong; correlations between radiographic views and full-length standing anteroposterior radiography were not determined; authors concluded that assessing leg length discrepancy using pelvic radiograph could not be recommended to determine the true preoperative leg length discrepancy |
|  |  |  |  |  |
